# Strategies for aggregating gene expression data: The collapseRows R function

**DOI:** 10.1186/1471-2105-12-322

**Published:** 2011-08-04

**Authors:** Jeremy A Miller, Chaochao Cai, Peter Langfelder, Daniel H Geschwind, Sunil M Kurian, Daniel R Salomon, Steve Horvath

**Affiliations:** 1 Interdepartmental Program for Neuroscience, UCLA, Los Angeles, California, USA; 2 Human Genetics Department, UCLA, Los Angeles, California, USA; 3 Biostatistics Department, UCLA, Los Angeles, California, USA; 4 Neurology Department, UCLA, Los Angeles, California, USA; 5 Department of Molecular and Experimental Medicine, The Scripps Research Institute, La Jolla, California, USA

## Abstract

**Background:**

Genomic and other high dimensional analyses often require one to summarize multiple related variables by a single representative. This task is also variously referred to as collapsing, combining, reducing, or aggregating variables. Examples include summarizing several probe measurements corresponding to a single gene, representing the expression profiles of a co-expression module by a single expression profile, and aggregating cell-type marker information to de-convolute expression data. Several standard statistical summary techniques can be used, but network methods also provide useful alternative methods to find representatives. Currently few collapsing functions are developed and widely applied.

**Results:**

We introduce the R function collapseRows that implements several collapsing methods and evaluate its performance in three applications. First, we study a crucial step of the meta-analysis of microarray data: the merging of independent gene expression data sets, which may have been measured on different platforms. Toward this end, we collapse multiple microarray probes for a single gene and then merge the data by gene identifier. We find that choosing the probe with the highest average expression leads to best between-study consistency. Second, we study methods for summarizing the gene expression profiles of a co-expression module. Several gene co-expression network analysis applications show that the optimal collapsing strategy depends on the analysis goal. Third, we study aggregating the information of cell type marker genes when the aim is to predict the abundance of cell types in a tissue sample based on gene expression data ("expression deconvolution"). We apply different collapsing methods to predict cell type abundances in peripheral human blood and in mixtures of blood cell lines. Interestingly, the most accurate prediction method involves choosing the most highly connected "hub" marker gene. Finally, to facilitate biological interpretation of collapsed gene lists, we introduce the function userListEnrichment, which assesses the enrichment of gene lists for known brain and blood cell type markers, and for other published biological pathways.

**Conclusions:**

The R function collapseRows implements several standard and network-based collapsing methods. In various genomic applications we provide evidence that both types of methods are robust and biologically relevant tools.

## Background

Genomic and other high dimensional data analyses often face the challenge to collapse multiple variables. Such collapsing of data can be advantageous for several reasons: 1) to allow direct comparison of similar data from unique sources, 2) to amplify the signal by removing noisy information, or 3) to reduce the computational burden. As a first example, consider the task of combining or contrasting gene expression data measured on different microarray platforms. Most array platforms measure multiple probes per gene, and different platforms typically include different probes for the same gene. To compare such expression data sets at the level of a gene identifier (e.g. Entrez ID or gene symbol) requires that the multiple probe measurements of a given gene be collapsed into a single gene measurement. An important, yet relatively unexplored, empirical question is how to choose the best representative probe. As a second example, consider the task of representing co-expression modules (clusters of genes) by a single value (referred to as a module centroid). Several approaches for finding a module centroid have been proposed in the literature (e.g. forming the mean, choosing the first principal component, etc), but it is unclear in which situation each method should be used. As a third example, consider the task of estimating cell type abundances based on data for lineage and cell-specific markers such as selectively expressed genes or proteins. Often, microarray analyses are performed using heterogeneous tissue (e.g., brain sections, whole blood); thus, gene expression changes occurring between two groups of samples could either be due to changes in transcriptional levels in one or more cell types, or they could be due to changes in cell type proportion variation. By accurately estimating cell type abundances including data from independent measurements using complementary technologies (e.g. flow cytometry, histology), more accurate measures of transcriptional changes could be established.

Statistically speaking, all of these tasks involve summarizing several closely related variables into one collapsed variable. Collapsing approaches fall into one of two categories: 1) composite values, which involve forming an average (often weighted) of multiple variables in a group; and 2) representatives, where a single variable is chosen from those in the group. Two examples of composite values include the mean and the first principal component (PC). The first PC explains the maximum amount of variation underlying the variables. For example, when summarizing the gene expression profiles of a co-expression module, the first PC is known as the module eigengene (ME; e.g. [1]). Several strategies can be used for choosing a representative; for example, one could choose the variable with the least number of missing data or the maximum mean expression value or the maximum variance. Apart from these statistical approaches, network methods are increasingly used to collapse data. For example, one can first construct a network between the variables and then choose the one that has the highest connectivity (or degree; see the Methods section for more details). Toward this end, signed correlation networks are particularly relevant since often one wants to keep track of the direction of the relationship between variables. For example, in the case of transcriptional networks, genes showing positive correlation are more likely than uncorrelated or negatively correlated genes to have known protein-protein interactions [2-4].

In the present work, we have created an R function, collapseRows, that implements a host of widely used collapsing methods. We will describe this function in more detail and present several motivating examples involving gene expression data (mRNA abundances). We explore this function in the context of the several distinct gene expression-related situations presented above, describing situations when certain collapsing methods are advantageous. For example, we find that in most microarray experiments performed on brain tissue it is best to choose the probe with the maximum mean expression per gene, whereas when choosing a single gene to represent a co-expression module, the optimal collapsing method depends on the goal of the analysis. For predicting the true proportion of cell types within a tissue homogenate, on the other hand, network-based collapsing approaches perform best. Finally, we provide R code for both the collapseRows function as well as for performing all of the analyses discussed in the text. Overall, we find that the collapseRows function is useful in many situations in which data aggregation is required.

## Results

**Figure 1:**
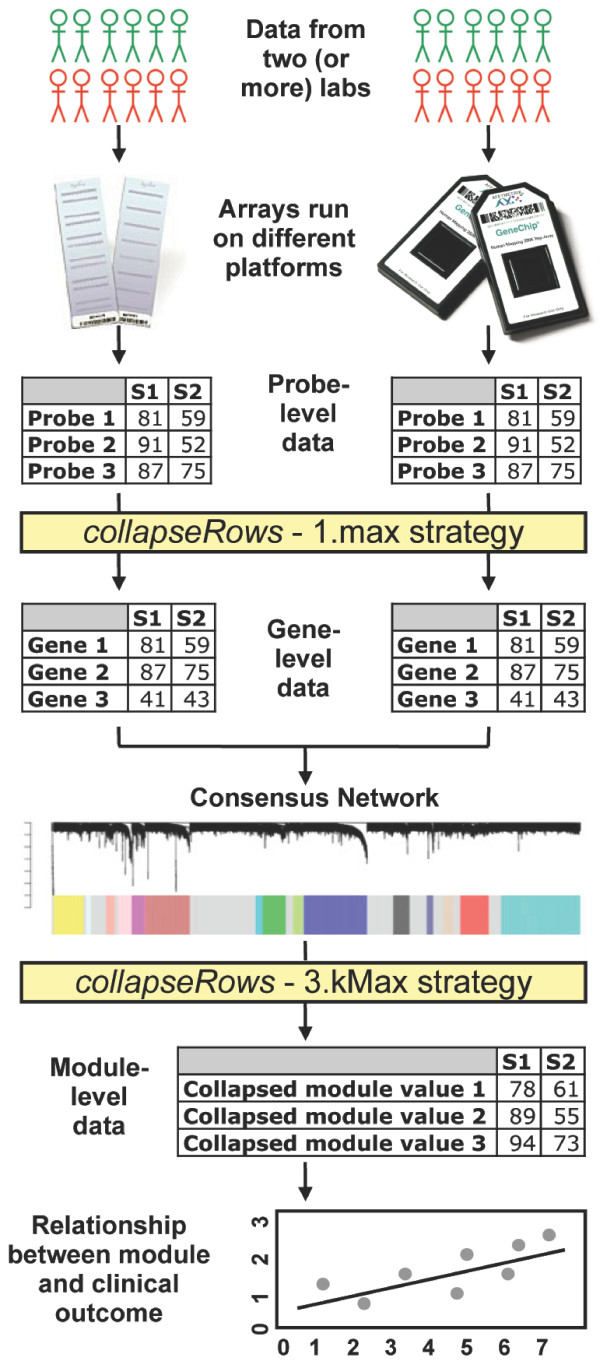
**Example pipeline for using collapseRows functions**. The collapseRows function could be used in two steps of a pipeline for finding predictors of a clinical outcome. First, probes could be collapsed into genes by taking the probe with the highest expression (1.max strategy) to allow comparability of data run using several microarray platforms (or RNAseq). These data could then be combined into a consensus module. Second, modules from the resulting network could be summarized using the the most highly connected gene (3.kMax strategy), some of which will likely be related to clinical outcomes.

Robust methods for summarizing large quantities of genomics data can be critical to advancing biological understanding. To address this issue we created collapseRows, which takes a numeric matrix of data as input, in which rows correspond to the variables, as well as a grouping variable that assigns each row to a group. This function implements standard collapsing methods (such as forming the average), as well as alternative network based methods (such as connectivity based collapsing) as possible strategies for data aggregation, then outputs one representative or composite value per group of rows. Thus, the resulting matrix has the same number of columns as the input matrix but typically has far fewer rows, potentially alleviating computational bottlenecks and often increasing between-study comparability. For example, in Figure 1 we present a hypothetical pipeline for using microarrays from multiple data sets to predict clinical outcome, in which collapseRows is used twice: first to collapse probes to genes, and then to collapse genes to modules.

In the following, we describe the R implementation of collapseRows and present examples from several empirical studies of high dimensional genomics data. In particular, for each of our examples, we run a subset of the following six collapsing strategies: maximum mean (1.max), maximum variance (2.var), maximum mean + maximum connectivity (3.kMax), maximum variance + maximum connectivity (4.kVar), module eigengene (5.ME), and the average (6.Avg; see Methods for details). Table 1 provides an overview of our empirical studies and the types of employed collapsing strategies.

**Table 1:** Summary of data sets and corresponding collapsing strategies

Fig	Analysis	Data sets used	1. max	2. var	3. kMax	4. kVar	5. ME	6. Avg
**1**	Summary	Hypothetical data	X	-	X	-	-	-

**2**	Collapsing probes to genes	18 Human Brain # 20 Mouse Brain % 5 Human Blood $	X	X	X	X	-	-

**3**	Choosing module centroids	7 Human Brain # 8 Mouse Brain % 5 Human Blood $	X	-	X	-	X	-

**4**	Predicting cell type proportions	Abbas et al 2009 (cell lines)	X	-	X	-	X	X

**5**	Predicting cell type proportions	Grigoryev et al 2010 (whole blood)	X	-	X	-	X	X

**Figure 2:**
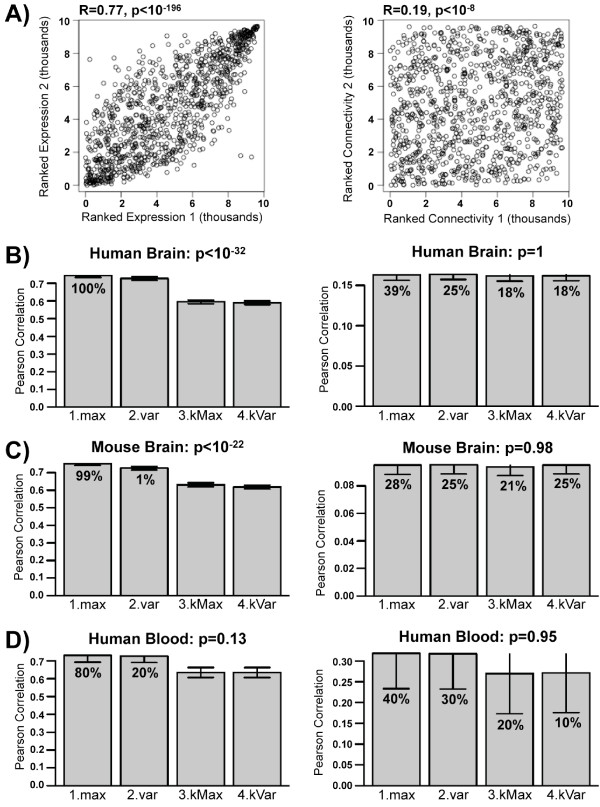
**When collapsing probes to genes, 1.max is usually the optimal collapsing strategy to choose**. A) A typical example of ranked expression (left column) and ranked connectivity (right column) correlation between two data sets. Each dot represents a gene in common between data sets, with the x and y axes represented that gene's ranked expression or connectivity in data sets 1 and 2, respectively. B-D) Across several studies in human brain (B), mouse brain (C), and human blood (D) the MaxMean (1.max) parameter generally produces better ranked expression correlations (left column) than maxVariance (2.var). For both MaxMean and maxVariance, use of connectivityBasedCollapsing (3.kMax and 4.kVar) decreases the between-study correlations. Similar results hold, to a lesser extent, with connectivity correlations (right column). Y-axes correspond to the average expression and connectivity correlation between data sets. Error bars represent standard error. Percentages indicate the percent of assessments in which the relevant strategy had the highest overall between-set correlation.

**Figure 3:**
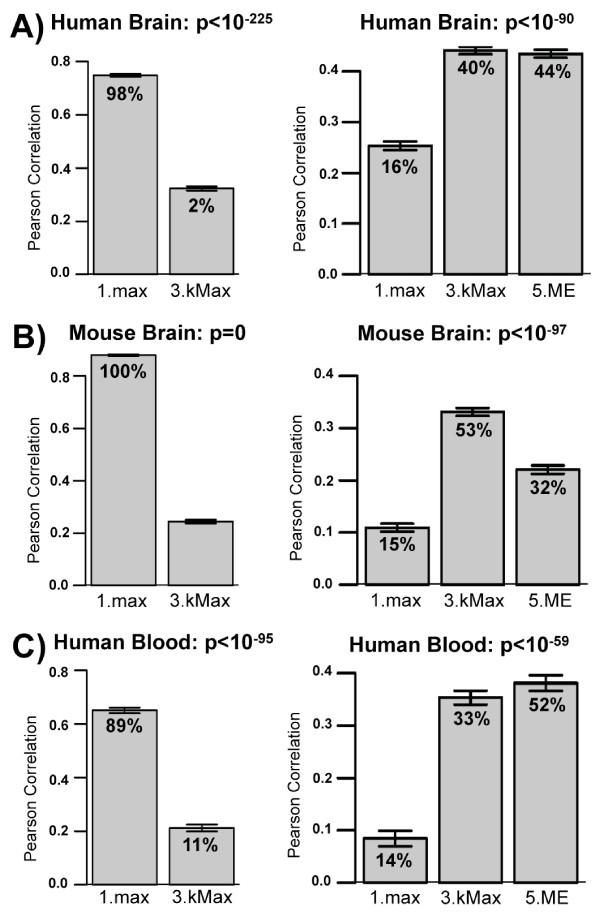
**When collapsing genes to modules, the optimal method depends on the goal of the analysis**. A-C) Across several studies in human brain (A), mouse brain (B), and human blood (C) the *MaxMean *parameter generally produces better ranked expression correlations (left column) when setting the *connectivityBasedCollapsing *parameter to FALSE. On the other hand, better connectivity correlations (right column) are found when setting *connectivityBasedCollapsing *to TRUE, in some cases producing higher correlations that even the module eigengene (5.ME). Labelling as in Figure 2 B-D.

"#" - The 18 human brain data sets were the following GSE numbers: 1133, 1297, 1572, 2164B, 3526A, 3526B, 3790A, 3790B, 3790C, 4036, 4757, 5281A, 5281B, 5388A, 5388B, 7621, 8397, and 9770. "%" - The 20 mouse brain data sets were the following GSE numbers: 1482, 1782A, 1782B, 2392, 3248, 3327A, 3327B, 3594C, 3963A, 3963B, 4269, 4734, 5429, 6285, 6514A, 6514B, 9444A, 9444B, 9444C, 10263. For "#" and "&," underlined data sets were used in Figure 3 as well as Figure 2. See Miller et al 2010 for more details on these data sets. "$" - The 5 human blood data sets were from Dumeaux et al 2010, Goring et al 2007, Pankla et al 2009, and Saris et al 2009.

### R implementation of the function collapseRows

This collapseRows function implements several network-based and biostatistical methods for finding a single value to represent each group specified in the function call. collapseRows is available in the WGCNA package for R [5], and is presented as Additional file 1: the collapseRows function, while code for performing all of the above analyses is presented in Additional file 2: R code to perform analysis. Accompanying data files can be downloaded from the collapseRows website [6]. Our code was run using version 2.12.2 of R, although any recent version should work. A few steps of the analysis (i.e., building the networks for example 2) require a computer with a large amount of RAM (a computer with 16 GB of RAM was used for these steps); however, most analysis steps can be performed on laptops (e.g. we used a MacBook Pro with 4 GB RAM in most analyses steps), and the entire analysis can be reproduced in a few hours. The R function is called as follows:

collapseRows(datET, rowGroup, rowID, method="MaxMean",

connectivityBasedCollapsing=FALSE, methodFunction=NULL,

connectivityPower=1, selectFewestMissing=TRUE)

In this call, *datET *represents a matrix or data frame containing numeric values where rows correspond to variables (e.g. microarray probes) and columns correspond to observations (e.g. microarrays). Each row must have a unique row identifier (specified in the vector *rowID*). The group label of each row is encoded in the vector *rowGroup *(e.g. gene symbols corresponding to the probes in *rowID*). The argument *method *is a character string for determining which method is used to collapse rows into groups. The implemented options, described in more detail in the Methods, are "MaxMean," "MinMean," "maxRowVariance," "absMaxMean," "absMinMean," "ME", "average," and "function." If *method *= "function", the method used is set by the argument *methodFunction*, which must be a function that takes a matrix of numbers as input and produces a vector the length of the number of columns as output (e.g., colMeans). The logical argument *connectivityBasedCollapsing *controls whether groups with 3 or more corresponding rows should be collapsed by choosing the row with the highest connectivity according to a signed weighted correlation network adjacency matrix (Methods), where the power is determined by the argument *connectivityPower*. All collapsing strategies presented in the empirical studies were formed by setting a combination of the *method *and *connectivityBasedCollapsing *parameters. Finally, the logical argument *selectFewestMissin*g (with default value TRUE), controls whether *datET *should be trimmed such that only the rows with the fewest number of missing values per group are retained. Detailed information regarding the function is available within R using the help(collapseRows) command.

### Example 1: collapsing microarray probes to genes

Gene expression microarrays often have several probes (or probe sets) per gene. Collapsing methods can be used to arrive at a single measurement per gene, which facilitates merging of data across different platforms. For example, in a previous study of mouse and human brain, a preliminary version of collapseRows was used to convert gene expression matrices from 18 human and 20 mouse data sets run on five separate microarray platforms into matrices that could be compared [3] (see Methods). Here we re-analyze these same data sets, along with several additional data sets from studies of human blood [7-12].

We evaluate the performance of the collapsing methods in multiple ways. To begin with, we determine how reproducible the result of the collapseRows function is across different data sets. For example, assume two different gene expression data sets in brain are available. After collapsing the probes by gene one can calculate the mean expression value of each gene in the two data sets. Next one can correlate the mean expression levels of the first data set with those of the second data set. A high correlation indicates that the output of the collapsing method allows one to robustly define the mean expression level of a gene. Since we are mainly interested in ranked expression levels, we determine the correlation of ranked mean expressions (which amounts to using the Spearman correlation coefficient). Apart from studying the reproducibility of the mean expressions, we also study the reproducibility of the network connectivity since this informs co-expression network applications. The connectivity (or degree) is the most widely used network statistic for describing the topological properties of a network node (gene) [13]. It is of great practical interest to determine to which extent the connectivity (and hub status) is preserved between 2 data sets. For example, it has often been used to compare networks from different species [3,14]. In this case, we define a signed weighted correlation network (Methods) after collapsing the probes into genes. Next we calculate the whole network connectivity measure k_i_, which measures how correlated the i-th gene is with other genes in the network (Methods). A significant positive correlation of ranked connectivity suggests that a co-expression network between the genes (collapsed probes) is robustly defined.

To empirically assess the reliability of different strategies of probe selection, we ran the collapseRows function on the brain and blood data sets described above, using 1.max, 2.var, 3.kMax, and 4.kVar. For each pair of gene expression matrices, we took the subset of genes in common and correlated the ranked mean expression and the ranked connectivity between data sets for each of the four collapsing strategies (see Figure 2A for a typical example; while the scatter plots do not reveal a strong relationship, we should point out that the connectivity correlations are significantly different from zero and non-negligible - at p < 10^-8 ^the correlation coefficient is highly significantly different from zero). We then determined the average correlation of these two measures across data set pairs in human brain (Figure 2B), mouse brain (Figure 2C), and human blood (Figure 2D) and assessed how frequently and to what degree these measures were highest for each collapsing strategy. Overall, we found that 1.max was the most robust method, particularly with regards to the expression correlation; in other words, choosing the probe with the highest mean expression nearly always lead to the most comparable results between these data sets. We should point out that numerous alternative approaches for collapsing probes per gene are possible. Some of them are discussed below.

### Example 2: choosing one centroid per gene expression module

The collapseRows function allows one to aggregate the genes that make up a co-expression module. A co-expression module is sometimes defined via a clustering method, but any module assignment can be specified with the row-grouping variable of the collapseRows function (also see examples 3 and 4 below). We assessed the reliability of the following three different strategies for collapsing module genes into a single representative per module: 1.max, which chooses the module gene with highest mean expression; 3.kMax, which chooses the most highly connected intramodular hub gene; and 5.ME, which defines the first principal component of the module genes, resulting in the module eigengene. We focused only on these three collapsing methods since they are often used in network based data reduction methods [13].

To perform this analysis, we started with expression data from a single data set in each group (GSE3790B for human brain, GSE4734 for mouse brain, and [9] for human blood) and defined co-expression modules using WGCNA, since it is a widely used method for defining co-expression modules [3-5,15-19]. Modules were defined as branches of a hierarchical clustering tree. To increase the robustness of our results, different choices of branch cutting parameters implemented in the dynamicTreeCut R library [20] were chosen to define 28 different module assignments. For each choice of module assignments (grouping variables), we then ran collapseRows on the subset of seven human and eight mouse brain data sets run on the HG-U133A and MG-U430A Affymetrix platforms, respectively, and on all five human blood data sets from example 1. Finally, we calculated the resulting ranked mean expression and ranked connectivity correlations between the different data sets as described above (Figure 3). If rows represent genes in a module, the connectivity of the collapsed variables measures how closely related the module is to other modules. Several studies have found that these correlations between modules may reveal higher order relationships between them [1,3,4].

For human brain (Figure 3A), mouse brain (Figure 3B), and human blood (Figure 3C) the ranked mean correlation measure suggests that 1.max is the best collapsing strategy, whereas the ranked connectivity measure suggests that 3.kMax is a better collapsing strategy. Furthermore, in the case of mouse brain, 3.kMax actually leads to higher ranked connectivity correlations than the standard method of calculating the module eigengene (5.ME). Together, these results suggest that different parameter options should be chosen depending on the goal of the analysis and the specific input data being used. For example, if the goal of the analysis were to determine correlation patterns between modules then 3.kMax would be a more appropriate collapsing strategy than 1.max.

### Example 3: predicting cell type abundances in mixtures of pure cell lines

Most microarrays measure tissue RNA from tissue homogenate rather than from a pure cell type. It has been recognized that markers for pure cells can sometimes be used to estimate the proportion of pure cells that make up the tissue homogenate. For example, standard expression deconvolution (based on a multivariate regression model) can be used to simultaneously estimate the abundance of multiple cell types [7,21-23]. While using a multivariate linear model may afford greater efficiency in estimating cell type abundances, it may perform poorly if it is incorrectly specified, for example due to omission of an unknown cell type. Our collapseRows based approach to expression deconvolution addresses a more limited, but related question: how well can expression levels of a set of marker genes for a single cell type (group) be used to estimate the relative abundance of that cell type between samples? Thus, the rows of the input matrix correspond to marker genes and the grouping variable assigns markers to their respective cell types. The collapsed variable per group is used as an abundance measure for the pure cell line.

To evaluate the performance of expression deconvolution methods, Abbas and colleagues [7] created a data set that involved mixing four distinct "pure" cell lines--Raji and IM-9 (from B cells), Jurkat (from T-cells), and THP-1 (from monocytes)--using pre-specified proportions. These known proportions could be used as gold standard measurement for judging the performance of methods that aim to estimate cell type abundances. Included in the expression data are microarrays run on each pure cell line, as well as microarrays run on four mixtures of these cell lines in known proportions, each in triplicate [7].

We applied the collapseRows function to the microarray data from the cell mixture study described above. We first chose the top 500 marker genes for each of the four cell lines by finding the genes with the highest fold change enrichment in a given cell line compared with the other three. We then ran the collapseRows function on the four mixtures of cell lines, using these 2000 marker genes as rows and the four cell lines as groups, and using several collapsing methods. Finally, we scaled these collapsed expression matrices to obtain the predicted proportions of each cell type across samples. We should emphasize that a limitation of our deconvolution approach is that it can only estimate the proportion of cells up to a constant. While our prediction is expected to be correlated with the true proportion across samples, it cannot be used to estimate the true proportion. The situation is similar to predicting degrees Fahrenheit based on another temperature scale: 0 degrees Fahrenheit does not correspond to 0 degrees Celsius, but a linear transformation can relate Fahrenheit to Celsius. It is often sufficient to use such an approach in the context of measuring cell type abundance, e.g. when correlating cell type abundances to a clinical outcome, because the correlation measure is scale invariant.

**Figure 4:**
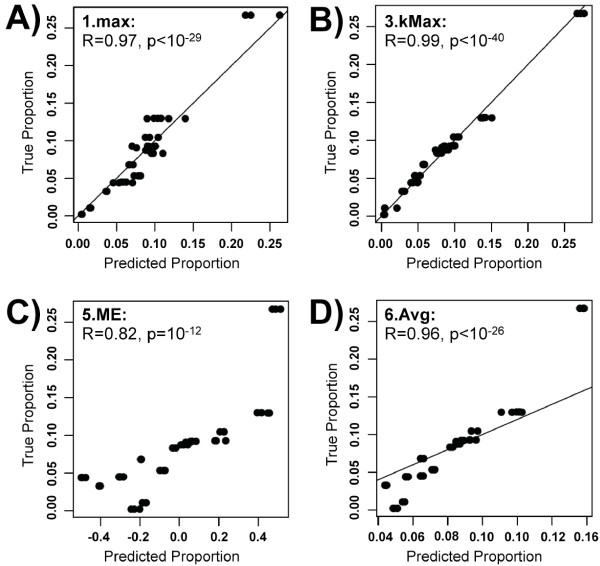
**collapseRows accurately predicts the relative quantity of blood cell lines across mixed samples**. Four prediction methods were used on data from (Abbas et al 2009), from which both gene expression data and actual blood cell counts were known: A) maximum mean expression (1.max), B) maximum connectivity (3.kMax), C) module eigengene (5.ME), and D) average (6.Avg) expression of all marker genes. Each dot presents one cell type in one sample. The X-axes correspond to the predicted proportion of each cell type, while the Y-axes correspond to the actual proportion of each cell type across samples. Values are scaled so that the sum of the proportions for a single cell type across all samples is 1. For all methods (except ME), the x = y line (representing perfect agreement) is plotted. Note that choosing the gene with the highest connectivity (B) most accurately predicts the true cell type proportions.

To assess the performance of different marker gene collapsing methods, we correlated the predicted abundances (proportions) of pure cell types with the true proportion determined by the mixture proportions (Figure 4). We find that all four tested methods of collapsing genes--1.max, 3.kMax, 5.ME, and 6.Avg--produced significant correlations (R > 0.8), but that collapsing based on maximum connectivity (3.kMax) led to by far the most accurate predictor (R = 0.994). In this application, collapsing rows by choosing the module eigengene (5.ME) leads to the least accurate predictions.

### Example 4: Predicting cell type abundances in peripheral human blood

**Figure 5:**
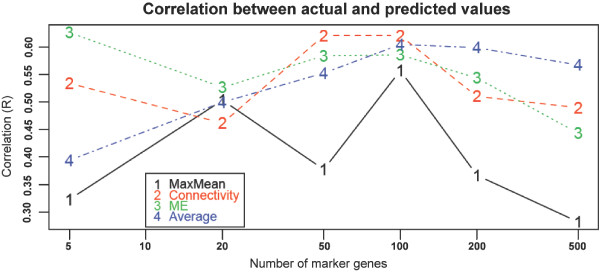
**collapseRows accurately predicts the relative quantity of cell type across samples of whole blood**. Using data from a realistic blood model (Grigoryev et al 2010), the 1.max, 3.kMax, 5.ME, and 6.Avg collapseRows aggregation strategies can still predict the relative proportion of several major cell types. Each point represents the correlation between true and predicted proportions for one of the four strategies. The X-axis corresponds to the number of marker genes used for the predictor, while the Y-axis corresponds to the correlation between true and predicted proportions. Note that all methods other than *MaxMean *(1.max) are relatively robust to choice in number of marker genes.

Finally, to assess whether these methods can accurately predict cell type abundances in a more realistic model, we obtained microarray data from a deconvolution study of peripheral blood both pre- and post-kidney transplant [10]. Similar to the previous data set, these data include microarrays run on pure cell populations (CD4+ and CD8+ T cells, and CD19+ B cells) as well as on whole blood; however, these arrays were run using actual human blood and the constituent blood cell populations were purified using magnetic beads from the same samples used for whole blood assays. To assess the performance of different marker gene collapsing methods, we correlated the predicted abundances (which up to a constant are proportions) of pure cell types with the true proportion determined by flow cytometry data (described in [10]). Again, we find high correlation between true and predicted cell proportions for these cell types (Figure 5; 0.5 < R < 0.6 in most cases), although unsurprisingly, these real cell subset predictions for whole blood are not as accurate as the highly contrived strategy of using fixed combinations of transformed cell lines by Abbas and colleagues [7]. We also varied the number of marker genes per cell type used to form predictor. Collapsing on connectivity (3.kMax), ME (5.ME), and average (6.Avg) all lead to comparably good predictors. Furthermore, choosing N~100 marker genes produces the best results, although these three predictors are all fairly robust to the number of marker genes chosen. Choosing the gene with the maximum mean expression (1.max), however, is a much less robust method, and should not be used in this situation.

### Data aggregation using *collapseRows *leads to increased reproducibility

Despite advantages, data aggregation or collapsing will inevitably lead to a loss of information. Therefore, it is important to assess whether the loss of information has an impact on analysis results and if so what the impact looks like. Most of our empirical analyses use reproducibility (as measured by correlation of ranked mean expression and connectivity) as a measure of how much usable information remains in the data after aggregation. We now extend these analyses to assess how selective removal of information using collapseRows affects reproducibility, using the subset of 7 human brain samples run on the HG-U133A array and the subset of 8 samples run on the MG-U430A array. We measured the correlation of ranked mean expression and connectivity for comparisons on the probe level, and compared those with our best result using collapseRows (1.max; see Additional file 3 - Increase in reproducibility using collapseRows). In human we find average expression correlations of R = 0.87 and 0.87, and connectivity correlations of R = 0.23 and 0.33 for the probe-level and gene-level comparisons, respectively. In mouse the comparable correlations are R = 0.93 and 0.93, and R = 0.15 and 0.24 for probe-level and gene-level comparisons. Together, these results suggest that, while present, the loss of information using collapseRows actually leads to increased reproducibility, at least as measured by correlation of ranked connectivity.

### The function userListEnrichment facilitates biological interpretation of gene lists

After collapsing gene lists into groups, either for the purpose of finding co-expression modules or for determining marker genes, it is often useful to relate the collapsed data to cell type markers. For example, when using collapseRows to predict cell type abundances in a data set that does not contain control samples from pure cell lines, it would be useful to be able to compare each gene group against lists of known marker genes for the expected cell type before using these groups for deconvolution. Such a biological indicator is particularly useful when studying brain, where it is almost never possible to precisely separate cell types as an experimental control. To address this issue, we have created the function userListEnrichment, which is also available in the WGCNA library [5]. This function measures enrichment of input lists of genes (i.e., groups found using collapseRows) with respect to pre-defined or user defined collections of brain- and blood-related lists curated from the literature (see the help file for specific references cited). Files containing user-defined lists can also be input as reference lists directly. userListEnrichment measures enrichment using a hypergeometric test, after which significant enrichments are output to a file that can then be used for further biological assessments. While biological assessment of userListEnrichment is beyond the scope of this article, we present a tutorial for how to perform a co-expression analysis using both collapseRows and userListEnrichment on our website: http://www.genetics.ucla.edu/labs/horvath/CoexpressionNetwork/JMiller/.

## Discussion

The collapseRows function implements widely used collapsing methods and lesser known ones based on correlation network methodology. For example, we describe situations when it can be useful to collapse a group of numeric variables by determining the most highly connected hub variable. The main contributions of this article are: i) to describe important uses of this function, ii) to empirically compare different collapsing methods, and iii) to make recommendations regarding when to use various collapsing methods. Overall, our empirical studies show that collapseRows is a useful function in many situations when comparing data from separate high-throughput gene expression sources, with a set of default parameters that are often, but not always, the best parameters to use. In the case of collapsing probes to their respective gene symbols, for example, we find that the 1.max strategy (implemented by setting method = "MaxMean" and connectivityBasedCollapsing = FALSE) produces the most robust results. On the other hand, when using the collapseRows function to predict cell type abundances, we find that the 3.kmax strategy (implemented by setting connectivityBasedCollapsing = TRUE) leads to the most accurate results. In the more general case of choosing a single value as representative for all genes in a co-expression module, we would suggest trying multiple parameter options to see which work best for that particular set of data. Although originally designed and tested for expression data, in principle collapseRows could be used in any situation requiring data aggregation (e.g., fMRI data, methylation data, etc.). In this case, one should try several collapsing strategies to determine the most robust one for the task at hand.

For the special setting of expression data, alternative methods have been developed to aid in the annotation and comparison of data sets between studies. First, several sequence-based strategies for reannotating microarray probe identifiers based on updated genomic sequences have been proposed [24-27]. The most extensive such endeavour is the Array Information Library Universal Navigator (AILUN), which provides an up-to-date mapping between probe identifiers and Entrez gene IDs for microarray platforms in 79 species [25]. While very useful, these studies address a slightly different issue than the collapseRows function, improving probe annotations rather than combining similarly annotated probes. In fact, we expect that first downloading the appropriate data from AILUN and then running collapseRows would lead to an improvement in the robustness of one's results. Other software packages have sought to combine these steps to some degree. GeneCruiser, for example, allows users to find gene annotation, as well as display heat maps of data, view the location of probes in the genome using the UCSC Genome Browser, and perform keyword searches for probes [28], but it only includes Affymetrix data sets. Integrative Array Analyzer (iArray) allows the user to perform several between- and within-study analyses, including data processing, co-expression analyses, differential expression analyses, and graphical visualization [29], but requires genes from different array platforms to already be linked (i.e., collapsed) by gene name. In short, while all of these studies provide useful resources for gene expression analysis, the collapseRows function is involved in an important processing step for between-study analysis that, to the best of our knowledge, has not been systematically addressed elsewhere.

Although our study provides a key step in the comparison of multiple numeric matrices, particularly with regards to gene expression analysis, it also has several limitations. First, our empirical studies only involve the use of a weighted correlation networks for finding a representative hub gene, when in fact other association networks (e.g. based on mutual information) could be used. Fortunately, close relationships exist between the seemingly countless statistical and network based approaches for constructing networks. Along the same lines, we choose intramodular hub genes using the connectivity (sum of adjacencies), while in principle other centrality measures could be used. Third, our strategy 6.Avg only uses a straight average, whereas an equally valid approach would be to form a weighted average based on other statistical or biological information. Fortunately, through the use of the methodFunction parameter, these or any other collapsing functions could be used with collapseRows as required. Finally, it is important to note that group assignments should be carefully checked (for example, by using AILUN [24]) before running collapseRows, as this function does not test whether annotations are correct.

## Conclusions

The collapseRows functions implements powerful and widely used methods for combining related variables. For example, collapseRows can be used to collapse probes to genes, to collapse genes to modules, or both. It can be used to choose an optimal cell marker gene. It can be used to aggregate dependent variables in such a way that computational memory requirements are greatly diminished. Our applications illustrate that the proposed collapsing strategies lead to robust, reliable results.

## Methods

Abstractly speaking, we study methods for collapsing the rows of a numeric matrix. The word "collapse" reflects the fact that the method yields a new matrix whose rows correspond to a subset of rows from the original input data. The function collapseRows implements several biostatistical and network-based methods for finding a representative row or composite value for each group specified in *rowGroup*, which are described in detail below. One of the advantages of this function is that it implements the following default settings, which have worked well in numerous applications: first, each group is represented by the corresponding row with the fewest number of missing data. Often several rows have the same minimum number of missing values (for example, if there is no missing data) and a representative must be chosen among those rows; in this case, the function chooses the remaining row with the highest sample mean. In the rare case when multiple rows have the same mean, then the function randomly chooses a representative row.

### Using human and mouse brain data sets to assess reproducibility

All samples from the 18 human and 20 mouse brain data sets were taken from brain tissue and were all run on Affymetrix platforms. Given that we explore the same tissue (albeit from different species) we hypothesized that a subset of genes would show reproducible mean expression levels and network connectivity levels. For example, regardless of the experimental paradigm, there will be a subset of marker genes in neurons and glial cells that will be present, and this subset of genes should be reproducible. In a prior publication we confirmed that both mean expression and connectivity showed a significant amount of reproducibility in all of these data sets, even when comparing across species [3]. For example, the mean expression (Spearman correlation of R = 0.60, p < E-400) and connectivity (R = 0.27, p < E-70) was highly correlated between human and mouse brains. We and others have used the (Spearman) correlation to assess reproducibility of means and connectivities in pairs of data sets but we should point out that other measures are possible.

### Biostatistical methods for collapsing rows

The collapseRows function implements several standard methods for collapsing rows into their respective groups. First, the row with the highest mean value ("MaxMean") can be selected for each group (the default). Similarly, the representative row with the lowest mean value ("MinMean"), or the one with the highest or lowest mean absolute value ("absMaxMean" and "absMinMean," respectively) can also be chosen. Further, one can also select rows according to their variance across observations ("maxRowVariance"). In addition, the function implements two composite value methods: 1) the group value can be summarized as the average value ("average") across rows in a group for each column, and 2) the group value can be summarized as the first principal component of the rows in each group (referred to as the "ME" method since, for co-expression module applications, this amounts to calculating the module eigengene). Finally, collapseRows allows the user to input their own user-defined aggregation function (by setting *method *= "function" and passing the desired function using *methodFunction*), which affords maximum flexibility.

### Network methods for collapsing rows

Correlation network methods for collapsing variables are only meaningful when dealing with at least 3 variables (that have the same minimum number of missing values)--for groups with exactly two rows one of the above biostatistical methods is used for data aggregation ("MaxMean" by default). For groups of three or more rows, the collapseRows function constructs a signed weighted correlation network between the variables. A network is specified by the connection strengths, or *adjacencies*, defined for each pair of variables x_*i *_and x_*j *_and denoted by *a*_*ij*_. A mathematical constraint on *a*_*ij *_is that its values must lie between 0 and 1.

Weighted gene co-expression network analysis (WGCNA) [5,14] defines the signed weighted correlation network between *x*_*i *_and *x*_*j *_as:

where the power β is used as a soft threshold [14]. The advantage of using a signed network is that it preserves the sign of the underlying correlation coefficient. Note that a correlation of -1 leads to an adjacency of 0, while a correlation of 1 leads to an adjacency of 1. Soft thresholding (using the power β) preserves the continuous nature of the correlation information; alternative approaches based on hard thresholding the correlation coefficient may lead to information loss. While any power β could be used, the default power of the collapseRows function is β = 1 since in many applications relatively few rows correspond to one group. In this case, there is less of a need to threshold the correlation measure.

The *connectivity *(*k*) of the *i*-th node is defined by:

In weighted networks, the connectivity equals the sum of connection weights between node *i *and all other nodes in the network. Variables with high connectivity tend to be highly positively correlated with other variables. Connectivity based collapsing chooses the most highly connected row (highest k) as representative, and can be implemented with collapseRows by setting *connectivityBasedCollapsing *= TRUE. In this case, if several probes have the same maximum connectivity, the first such row is chosen.

### Collapsing methods used in the empirical studies

For each of our examples, we ran a subset of the following six collapsing methods and studied how often each method leads to the most reproducible results. First, we choose the row with the highest mean expression (1.max) by setting *method *= "MaxMean" and *connectivityBasedCollapsing *= FALSE. Second, we choose the row with the highest between-column variability (2.var) by setting *method *= "maxRowVariance" and *connectivityBasedCollapsing *= FALSE. Third, we choose the row with the highest connectivity in cases with three or more rows per group or highest mean expression in cases with two rows per group (3.kMax) by setting *method *= "MaxMean" and *connectivityBasedCollapsing *= TRUE. Fourth, we choose the row with the highest connectivity in cases with three or more rows per group or maximum variability in cases with two rows per group (4.kVar) by setting *method *= "maxRowVariance" and *connectivityBasedCollapsing *= TRUE. Fifth, as a sort of control, we compare our results to a standard method of centroid determination by measuring the module eigengene (first principal component) for all rows in a given group across all groups (5.ME) by setting *method *= "ME". Finally, in our assessment of blood cell type, we also use the average of all marker genes (6.Avg) by setting *method *= "average".

## Competing interests

The authors declare that they have no competing interests.

## Authors' contributions

JAM wrote the collapseRows function, carried out the analyses using the human and mouse brain data sets, and drafted the manuscript. CC carried out most of the analyses using human blood. PL tested the function in several settings and implemented it in the WGCNA package. DHG participated in the design of the human and mouse brain analyses, and provided critical input in the creation of the collapseRows function. DRS and SMK performed the sample collection, arrays, and flow cytometry analysis for the peripheral blood cell subset analysis and contributed to design and coordination of this aspect of the present study. SH participated in the design and coordination of the study, created initial R code for several aspects of the study, and helped to draft the manuscript. All authors read and approved the final manuscript.

## Supplementary Material

Additional file 1 **the collapseRows function**. This file contains the current version of collapseRows at the date of publication. The most current version of collapseRows is available as part of the WGCNA package [5]. Click here for file

Additional file 2 **R code to perform analysis**. This file includes all of the code required to reproduce Figures 2, 3, 4, 5 in this manuscript (and Additional file 3 - Increase in reproducibility using collapseRows), along with a limited amount of annotation. Data for use with this code is available at the collapseRows website [6]. Click here for file

Additional file 3 **Increase in reproducibility using collapseRows**. We calculated ranked expression (left column) and ranked connectivity (right column) correlation across 7 studies in human brain run on the HGu133A platform (A) and 8 studies in mouse brain run on the MGu430A platform (B). To assess change in reproducibility due to collapseRows, we compared these correlations using our best collapsing method (1.max) against uncollapsed data (0.None). We do not find any changes in reproducibility based on expression correlation; however, based on connectivity correlation we find a relatively substantial increase in reproducibility in both species. Note that these correlations are generally higher than the corresponding correlations from Figure 2 because we only show the correlations from data sets coming from the same platform. Y-axes correspond to the average expression and connectivity correlation between data sets. Error bars represent standard error. Click here for file
