# Supplementary material for: Strategies for aggregating gene expression data: The collapseRows R function
Source: BMC Bioinformatics. 2011 Aug 4;12:322. doi: 10.1186/1471-2105-12-322 (PMC3166942; doi:10.1186/1471-2105-12-322)

This document contains all of the code required to complete the analyes in Miller JA, Langfelder P, Cai C, Horvath S (2011) *Strategies for optimally aggregating gene expression data: The collapseRows R function.* Technical Report.

##############################
## CODE FOR MAKING FIGURE 2 ##
##############################

In this part of the meta-analysis we are going to try and determine similarities and differences between the mouse and human transcriptome in the brain and the human transcriptome in blood. All of the processing of the data sets has already been completed.

## Read in and format all of the files to R.
Note: the following section is the original code used to read in the data files. Since these files are too large, I have run this myself and saved everything as "startHMB.RData." The actual code starts at "START HERE" on the next page.

```
m2h = read.csv("mouse_human_orthology.csv")
rownames(m2h)=m2h[,2]
source("NetworkFunctions3.txt")
source("Jeremy_Functions_All.R")
library(WGCNA)
# source("collapseRows_04_11_11.R") ## NOT REQUIRED IF NEWEST VERSION OF WGCNA LIBRARY IS INSTALLED ##

# Human brain data
arraysH=c("1133","1297","1572","3526A","3526B","4036","4757","5281A","5281B","5388A","5388B","9770","2164B",
          "3790A","3790B","3790C","7621","8397")
fileNamesH=paste("HM_expression/GSE",arraysH,"_present_expression.csv",sep="")
dg = read.expression.data.as.list(fileNamesH)
dataH = dg[[1]]; genesH = dg[[2]]; rm(dg)

# Mouse brain data
arraysM=c("1482","1782A","1782B","2392","3248","3327A","3327B","3594C","3963A","3963B","4269","4734","5429",
          "6285","6514A","6514B","9444A","9444B","9444C","10263")
fileNamesM=paste("HM_expression/GSE",arraysM,"_present_expression.csv",sep="")
dg = read.expression.data.as.list(fileNamesM)
dataM = dg[[1]]; genesM = dg[[2]]; rm(dg)

# Human blood data
allProbes<-allGenes<-NULL
pg=get.pg.from.annotation.file("Mouse430A_2.na25.annot.csv")
allProbes=c(allProbes,pg[[1]]);  allGenes=c(allGenes,pg[[2]])
pg=get.pg.from.annotation.file("MG_U74Av2.na25.annot.csv")
allProbes=c(allProbes,pg[[1]]);  allGenes=c(allGenes,pg[[2]])
allGenes = mouse2human2(allGenes,m2h)
pg=get.pg.from.annotation.file("HG-U133A_2.na25.annot.csv")
allProbes=c(allProbes,pg[[1]]);  allGenes=c(allGenes,pg[[2]])
pg=get.pg.from.annotation.file("HG_U95Av2.na25.annot.csv")
allProbes=c(allProbes,pg[[1]]);  allGenes=c(allGenes,pg[[2]])
pg=get.pg.from.annotation.file("HG-U133_Plus_2.na25.annot.csv")
allProbes=c(allProbes,pg[[1]]);  allGenes=c(allGenes,pg[[2]])

dataB = list()
load("SAFHS_1111_47289.RData")
datExpr[is.na(datExpr)]=0;  dataB[[1]]=datExpr;  allProbes=c(allProbes,an[,1]);  allGenes=c(allGenes,an[,2])
load("Ducth_380_36058.RData")
datExpr[is.na(datExpr)]=0;  dataB[[2]]=datExpr;  allProbes=c(allProbes,an[,1]);  allGenes=c(allGenes,an[,2])
load("GIFT_266_20589.RData")
datExpr[is.na(datExpr)]=0;  dataB[[3]]=datExpr;  allProbes=c(allProbes,an[,1]);  allGenes=c(allGenes,an[,2])
load("Chaussabel_67_30535.RData")
datExpr[is.na(datExpr)]=0;  dataB[[4]]=datExpr;  allProbes=c(allProbes,an[,1]);  allGenes=c(allGenes,an[,2])
load("NOWAC_304_18439.RData")
datExpr[is.na(datExpr)]=0;  dataB[[5]]=datExpr;  allProbes=c(allProbes,an[,1]);  allGenes=c(allGenes,an[,2])

rm(datExpr)
keep = !duplicated(allProbes)
allProbes=allProbes[keep]; allGenes=allGenes[keep]
PR = allProbes; GE = allGenes;
```

```
## Function to return a subset of datOut that only includes rows for genes with >=nProbe probes

subsetDat <- function (datOut, rowGroup, rowID, nProbe=2){
  names(rowGroup) = rowID
  ids    = rownames(datOut);
  group  = rowGroup[ids]
  tGroup = table(group)
  keepG  = sort(names(tGroup)[tGroup>=nProbe])
  keepP  = ids[is.element(group,keepG)]
  return(list(data=datOut[keepP,],group=keepG,ids=keepP))
}

## Only take the subset of Blood data sets that we are going to use, since they are VERY big matrices
# Note: we need all of the genes from the mouse and human brain data for the next figure, so we do not subset
them here.

kpB <- list()
for (i in 1:5)  kpB[[i]] = subsetDat(dataB[[i]],GE,PR,2)[[3]]
for (i in 1:5)  dataB[[i]]= dataB[[i]][kpB[[i]],]

## Save the file to read in to R
save.image("startHMB.RData")


################
## START HERE ##
################

load("startHMB.RData")
library(WGCNA)
# source("collapseRows_04_11_11.R") # NOT REQUIRED IF NEWEST VERSION OF WGCNA LIBRARY IS INSTALLED ##


## Run collapseRows to collapse PROBES to GENES using 4 separate methods:
# (1) maxMean (refered to as "1.max")
# (2) maxVariance (refered to as "2.var")
# (3) connectivity method w/ maxMean (refered to as "3.kMax")
# (4) connectivity method w/ maxVariance (refered to as "4.kVar")
# Note, this section will take a LONG TIME (likely 90+ minutes) to run.

dataH1<-dataM1<-dataB1<-dataH2<-dataM2<-dataB2<-dataH3<-dataM3<-dataB3<-dataH4<-dataM4<-dataB4<-list()
for (i in 1:18){
 dataH1[[i]] = collapseRows(dataH[[i]],GE,PR,"MaxMean")
 dataH2[[i]] = collapseRows(dataH[[i]],GE,PR,"maxRowVariance")
 dataH3[[i]] = collapseRows(dataH[[i]],GE,PR,"MaxMean",TRUE)
 dataH4[[i]] = collapseRows(dataH[[i]],GE,PR,"maxRowVariance",TRUE)
};
dataHH = list(dataH1,dataH2,dataH3,dataH4,dataH)
for (j in 1:20){
 dataM1[[j]] = collapseRows(dataM[[j]],GE,PR,"MaxMean")
 dataM2[[j]] = collapseRows(dataM[[j]],GE,PR,"maxRowVariance")
 dataM3[[j]] = collapseRows(dataM[[j]],GE,PR,"MaxMean",TRUE)
 dataM4[[j]] = collapseRows(dataM[[j]],GE,PR,"maxRowVariance",TRUE)
};
dataMM = list(dataM1,dataM2,dataM3,dataM4,dataM)
for (i in 1:5){
 dataB1[[i]] = collapseRows(dataB[[i]],GE,PR,"MaxMean")
 dataB2[[i]] = collapseRows(dataB[[i]],GE,PR,"maxRowVariance")
 dataB3[[i]] = collapseRows(dataB[[i]],GE,PR,"MaxMean",TRUE)
 dataB4[[i]] = collapseRows(dataB[[i]],GE,PR,"maxRowVariance",TRUE)
};
dataBB = list(dataB1,dataB2,dataB3,dataB4,dataB)
# save(dataHH,dataMM,dataBB,GE,PR,file="dataMHB_all.RData") # This save is optional.


## Determine which genes are from 2+ probes for each data set
# The reason we omit genes with only 1 probe in the analysis is because collapseRows does not do anything to
these probes, regardless of the method chosen.  (This has already been done with the blood data.)

kpHH <- kpMM <- kpBB <- NULL
for (i in 1:18) kpHH[[i]]=subsetDat(dataHH[[5]][[i]],GE,PR,2)[[2]]
for (i in 1:20) kpMM[[i]]=subsetDat(dataMM[[5]][[i]],GE,PR,2)[[2]]
for (i in 1:5)  kpBB[[i]]=subsetDat(dataBB[[5]][[i]],GE,PR,2)[[2]]


## Find the average ranked expression of the 18 human and 20 mouse brain and 5 human blood data sets for each of
the 4 methods.
# This is the data for making figure 2, parts B-D, column 1
```

```
rnkExprHH<-rnkExprMM<-rnkExprBB<-list()
for (k in 1:4){
 rnkExprHH[[k]] = list()
 for (i in 1:18) rnkExprHH[[k]][[i]] = rank(rowSums(dataHH[[k]][[i]][[1]])[kpHH[[i]]])
}
for (k in 1:4){
 rnkExprMM[[k]] = list()
 for (i in 1:20) rnkExprMM[[k]][[i]] = rank(rowSums(dataMM[[k]][[i]][[1]])[kpMM[[i]]])
}
for (k in 1:4){
 rnkExprBB[[k]] = list()
 for (i in 1:5)  rnkExprBB[[k]][[i]] = rank(rowSums(dataBB[[k]][[i]][[1]])[kpBB[[i]]])
}


## Determine all of the interarray correlations of ranked expression.
# This is the data for making figure 2, parts B-D, column 1

corHH <- corMM <- corBB <- list()
corHH[[1]]<- corHH[[2]]<-corHH[[3]]<-corHH[[4]]<- matrix(nrow=18,ncol=18)
corMM[[1]]<- corMM[[2]]<-corMM[[3]]<-corMM[[4]]<- matrix(nrow=20,ncol=20)
corBB[[1]]<- corBB[[2]]<-corBB[[3]]<-corBB[[4]]<- matrix(nrow=5, ncol=5)

for (k in 1:4) for (i in 1:18) for (j in 1:18){
  ci = rnkExprHH[[k]][[i]]; cj = rnkExprHH[[k]][[j]]
  ov = intersect(names(ci),names(cj))
  corHH[[k]][i,j]=cor(rank(ci[ov]),rank(cj[ov]))
}
for (k in 1:4) for (i in 1:20) for (j in 1:20){
  ci = rnkExprMM[[k]][[i]]; cj = rnkExprMM[[k]][[j]]
  ov = intersect(names(ci),names(cj))
  corMM[[k]][i,j]=cor(rank(ci[ov]),rank(cj[ov]))
}
for (k in 1:4) for (i in 1:5) for (j in 1:5){
  ci = rnkExprBB[[k]][[i]]; cj = rnkExprBB[[k]][[j]]
  ov = intersect(names(ci),names(cj))
  corBB[[k]][i,j]=cor(rank(ci[ov]),rank(cj[ov]))
}


## Find the average ranked overall connectivity of the 18 human & 20 mouse data sets for each of the 4 methods.
# This is the data for making figure 2, parts B-D, column 2.  We use a signed network with power=10.
# Note: This step of the code can take a while (likely 30+ minutes) to run.

# Instead of running this section, you can run the following line: load("rnkConn.RData")
rnkConnHH <- rnkConnMM <- rnkConnBB <- list()
for (k in 1:4){
 rnkConnHH[[k]] = list()
 for (i in 1:18){
  dat = t(dataHH[[k]][[i]][[1]][kpHH[[i]],])
  rnkConnHH[[k]][[i]] = rank(softConnectivity(dat, type="signed", power=10, verbose=0))
  names(rnkConnHH[[k]][[i]]) = colnames(dat)}
}
for (k in 1:4){
 rnkConnMM[[k]] = list()
 for (i in 1:20){
  dat = t(dataMM[[k]][[i]][[1]][kpMM[[i]],])
  rnkConnMM[[k]][[i]] = rank(softConnectivity(dat, type="signed", power=10, verbose=0))
  names(rnkConnMM[[k]][[i]]) = colnames(dat)}
}
for (k in 1:4){
 rnkConnBB[[k]] = list()
 for (i in 1:5){
  dat = t(dataBB[[k]][[i]][[1]][kpBB[[i]],])
  rnkConnBB[[k]][[i]] = rank(softConnectivity(dat, type="signed", power=10, verbose=0))
  names(rnkConnBB[[k]][[i]]) = colnames(dat)}
}


## Determine all of the interarray correlations of ranked connectivity.
# This is the data for making figure 2, parts B-C, column 2

corCHH <- corCMM <- corCBB <- list()
corCHH[[1]]<- corCHH[[2]]<-corCHH[[3]]<-corCHH[[4]]<- matrix(nrow=18,ncol=18)
corCMM[[1]]<- corCMM[[2]]<-corCMM[[3]]<-corCMM[[4]]<- matrix(nrow=20,ncol=20)
corCBB[[1]]<- corCBB[[2]]<-corCBB[[3]]<-corCBB[[4]]<- matrix(nrow=5, ncol=5)

for (k in 1:4) for (i in 1:18) for (j in 1:18){
  ci = rnkConnHH[[k]][[i]]; cj = rnkConnHH[[k]][[j]]
```

```
  ov = intersect(names(ci),names(cj))
  corCHH[[k]][i,j]=cor(rank(ci[ov]),rank(cj[ov]))
}
for (k in 1:4) for (i in 1:20) for (j in 1:20){
  ci = rnkConnMM[[k]][[i]]; cj = rnkConnMM[[k]][[j]]
  ov = intersect(names(ci),names(cj))
  corCMM[[k]][i,j]=cor(rank(ci[ov]),rank(cj[ov]))
}
for (k in 1:4) for (i in 1:5) for (j in 1:5){
  ci = rnkConnBB[[k]][[i]]; cj = rnkConnBB[[k]][[j]]
  ov = intersect(names(ci),names(cj))
  corCBB[[k]][i,j]=cor(rank(ci[ov]),rank(cj[ov]))
}
```

## Make the plots for figure 2, B-D, using the above data

```
pdf("Figure1_BCD_new.pdf",width=9,height=12)
par(mfrow=c(3,2))
l = sum(lower.tri(corHH[[1]])); N=NULL
dNames = c(rep("1.max",l), rep("2.var",l), rep("3.kMax",l), rep("4.kVar",l))
d=N; for (i in 1:4) d=c(d,corHH[[i]][lower.tri(corHH[[i]])])
verboseBarplot(d,dNames,main="Human Brain",xlab="",ylab="Pearson Correlation",cex=1.5)
d=N; for (i in 1:4) d=c(d,corCHH[[i]][lower.tri(corCHH[[i]])])
verboseBarplot(d,dNames,main="Human Brain",xlab="",ylab="Pearson Correlation",cex=1.5)

l = sum(lower.tri(corMM[[1]])); N=NULL
dNames = c(rep("1.max",l), rep("2.var",l), rep("3.kMax",l), rep("4.kVar",l))
d=N; for (i in 1:4) d=c(d,corMM[[i]][lower.tri(corMM[[i]])])
verboseBarplot(d,dNames,main="Mouse Brain",xlab="",ylab="Pearson Correlation",cex=1.5)
d=N; for (i in 1:4) d=c(d,corCMM[[i]][lower.tri(corCMM[[i]])])
verboseBarplot(d,dNames,main="Mouse Brain",xlab="",ylab="Pearson Correlation",cex=1.5)

l = sum(lower.tri(corBB[[1]])); N=NULL
dNames = c(rep("1.max",l), rep("2.var",l), rep("3.kMax",l), rep("4.kVar",l))
d=N; for (i in 1:4) d=c(d,corBB[[i]][lower.tri(corBB[[i]])])
verboseBarplot(d,dNames,main="Human Blood",xlab="",ylab="Pearson Correlation",cex=1.5)
d=N; for (i in 1:4) d=c(d,corCBB[[i]][lower.tri(corCBB[[i]])])
verboseBarplot(d,dNames,main="Human Blood",xlab="",ylab="Pearson Correlation",cex=1.5)
dev.off()
```

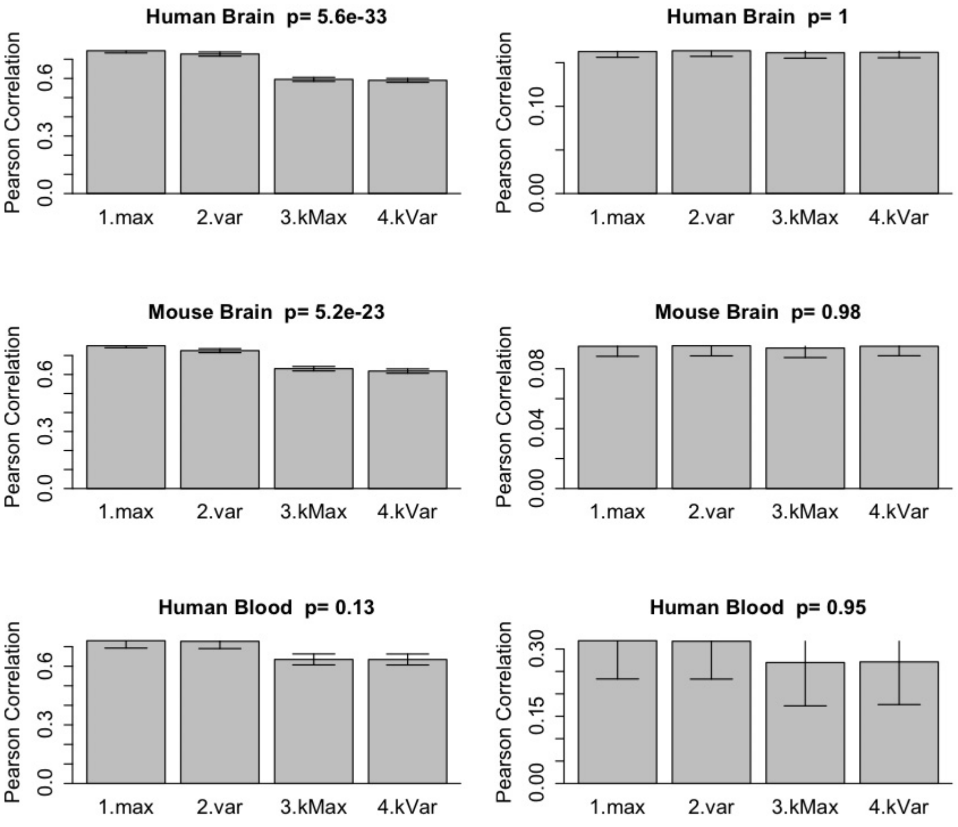

```
## Determine in what percentage of cases each of the methods is best.
# This is the code from which the percentages displayed in figure 1B-C are formed.

kh    = lower.tri(corHH[[i]]);
km    = lower.tri(corMM[[i]])
kb    = lower.tri(corBB[[i]])
allH  = cbind(corHH[[1]][kh],  corHH[[2]][kh],  corHH[[3]][kh],  corHH[[4]][kh])
allM  = cbind(corMM[[1]][km],  corMM[[2]][km],  corMM[[3]][km],  corMM[[4]][km])
allB  = cbind(corBB[[1]][kb],  corBB[[2]][kb],  corBB[[3]][kb],  corBB[[4]][kb])
allCH = cbind(corCHH[[1]][kh], corCHH[[2]][kh], corCHH[[3]][kh], corCHH[[4]][kh])
allCM = cbind(corCMM[[1]][km], corCMM[[2]][km], corCMM[[3]][km], corCMM[[4]][km])
allCB = cbind(corCBB[[1]][kb], corCBB[[2]][kb], corCBB[[3]][kb], corCBB[[4]][kb])
maxH  = apply(allH,1,which.max)
maxM  = apply(allM,1,which.max)
maxB  = apply(allB,1,which.max)
maxCH = apply(allCH,1,which.max)
maxCM = apply(allCM,1,which.max)
maxCB = apply(allCB,1,which.max)

round(100*table(maxH)/length(maxH))   # 100% maxMean
round(100*table(maxM)/length(maxM))   # 99% maxMean,  1% maxVar
round(100*table(maxB)/length(maxB))   # 80% maxMean, 20% maxVar
round(100*table(maxCH)/length(maxCH)) # 39% maxMean, 25% maxVar, 18% maxMean+conn, 18% maxVar+conn
round(100*table(maxCM)/length(maxCM)) # 28% maxMean, 25% maxVar, 21% maxMean+conn, 25% maxVar+conn
round(100*table(maxCB)/length(maxCB)) # 40% maxMean, 30% maxVar, 20% maxMean+conn, 10% maxVar+conn


## Plot an example of the correlations summarized above in the bar plots
# This is the data and plots for Figure 2A

d1 = dataH[[1]]; d2 = dataH[[2]]
ov = intersect(rownames(d1),rownames(d2))
d1 = d1[ov,]; d2 = d2[ov,]

rankExpr1 =rank(rowMeans(d1))
rankExpr2 =rank(rowMeans(d2))
rankConn1 =rank(softConnectivity(t(d1),type="signed",power=10))
rankConn2 =rank(softConnectivity(t(d2),type="signed",power=10))

pdf("Figure1_A.pdf")
par(mfrow=c(2,2))
verboseScatterplot(rankExpr1,rankExpr2,xlab = "Ranked Expression (1)", ylab = "Ranked Expression (2)")
verboseScatterplot(rankConn1,rankConn2,xlab = "Ranked Connectivity (1)", ylab = "Ranked Connectivity (2)")
s=sample(1:length(rankConn1),1000)
verboseScatterplot(rankExpr1[s],rankExpr2[s],xlab = "Ranked Expression (1)", ylab = "Ranked Expression (2)")
verboseScatterplot(rankConn1[s],rankConn2[s],xlab = "Ranked Connectivity (1)", ylab = "Ranked Connectivity (2)")
dev.off()
```

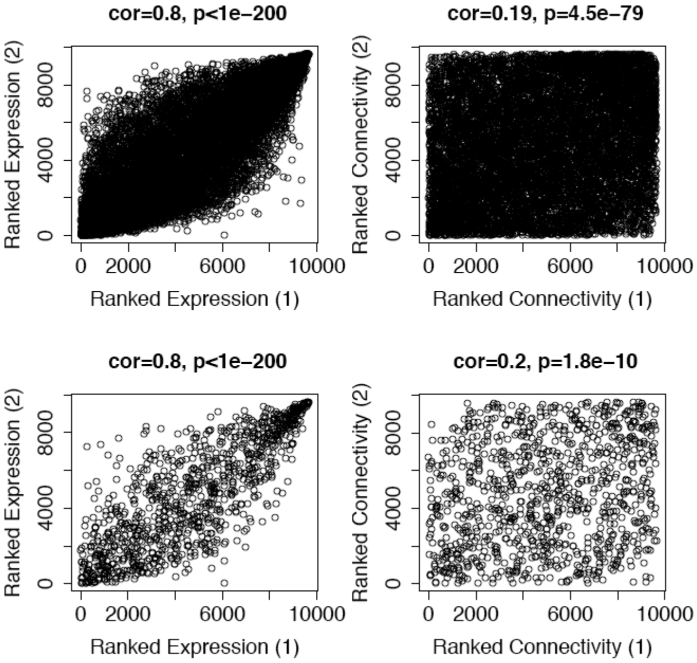


```
# The top row is all of the data, while the bottom row is a random sample of 1000 genes to display, for clarity.
The results you get for the bottom row may not be identical as Figure 1A or as the results displayed here, but
the correlations should be very close.
```

```
#####################################################
## CODE FOR MAKING FIGURE 3 (A-B: part C below) ##
#####################################################

# Note: This section uses the same data as the previous section

## Run an analysis comparing collapseRows methods when used to collapse GENES into MODULES of various sizes.
First, only look at the HUMAN data sets from HGu133A (Figure 2A)

platH  = c(2,2,1,3,3,3,3,3,3,2,2,3,1,2,2,2,3,2) # we want "2"(HGu133A)
dataNew = dataH[platH==2][2:8][c(5,6,2,3,4,7,1)]
   # The first data set was omitted due to too few genes. Remaining sets ordered by decreasing number of genes.
probes = rownames(dataNew[[1]])
for (i in 2:7) probes=intersect(probes,rownames(dataNew[[i]]))
length(probes) # [1] 10306
for (i in 1:7) dataNew[[i]] = dataNew[[i]][probes,]


# Now we have 7 comparable data sets.  The first one will be used as the base set in which modules will be made.
# Comment: In theory, any of these data sets could be used as base.

datExpr1= t(dataNew[[1]])
pickSoftThreshold(datExpr1[,sample(1:10306,7000)],networkType="signed",powerVector=c(6,8,9,10,12))
Power SFT.R.sq slope truncated.R.sq mean.k. median.k. max.k.
# 1      6    0.617 -5.13           0.937    200.0      195.0     345
# 2      8    0.815 -3.98           0.956     79.2       74.8     193
# 3      9    0.871 -3.60           0.957     51.7       47.9     152
# 4     10    0.912 -3.37           0.960     34.5       31.1     124
# 5     12    0.963 -2.98           0.978     16.3       13.8      87
# Choose power=9, since R.sq>0.8 and median.k>30 (power=8 or 10 would also have been okay)
# (Due to the sampling of 7000 genes, your results may not be identical. Also, this step above is optional.)

# Note: This section will take a little while (5-45 minutes, probably, and you need a powerful computer)
power = 9
AdjMatrest1 = adjacency(datExpr1,power=power,type="signed");
diag(AdjMatrest1)=0
dissTOM1 = 1-TOMsimilarity(AdjMatrest1, TOMType="signed");
geneTree1 = flashClust(as.dist(dissTOM1),method="average");
collectGarbage()


## Save various module-cutting parameters to use as tests for the collapseRows function.
# Note: This section will take a little while (5-30 minutes, probably)

pdf("collapseRowModuleColorsHuman.pdf", height=30,width=30);
par(mfrow=c(2,1), cex = 1.4, mar = c(0,8.5,2,0));
plot(geneTree1,labels=F,main="Hierarchical dendrogram & module colors",sub="",xlab="")
par(mar = c(1,8.5,0,0));
mColorh=NULL; labels=NULL
for (ds in 0:3) for (mc in c(4,8,16,32,64,128,256)){
 tree = cutreeHybrid(dendro = geneTree1, pamStage=FALSE,
   minClusterSize = mc, cutHeight = 0.99,
   deepSplit = ds, distM = dissTOM1)
 mColorh=cbind(mColorh,labels2colors(tree$labels));
labels = cbind(labels,paste("Deepsplit =",ds,"; MinSize =",mc))
}
plotHclustColors(geneTree1,mColorh,labels,main = "");
dev.off()
```

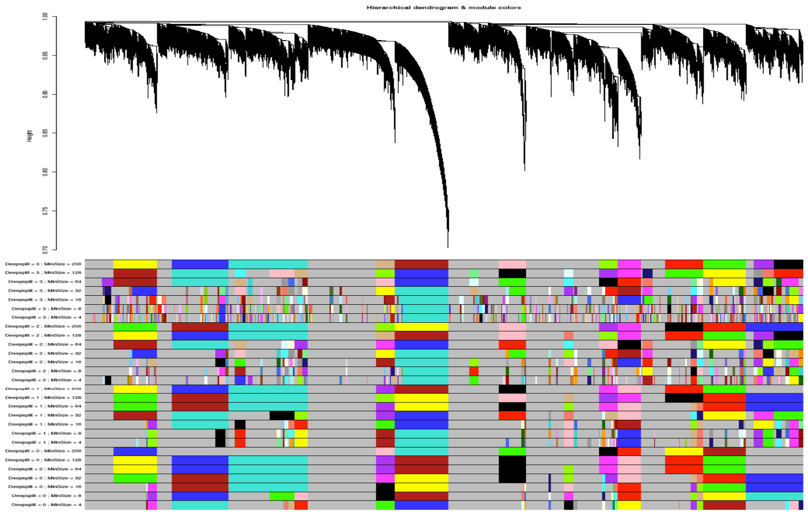

```
## Now run collapseRows a bunch of times, and see which of 3 methods does it best for various module schemas:
# (1) maxMean (refered to as "1.max")
# (2) connectivity method w/ maxMean (refered to as "3.kMax")
# (3) choosing the module eigengene (refered to as "5.ME")
# This step also will take a while (10-45 minutes)

dataHM<-dataHC<-MEs<-list(D1=list(),D2=list(),D3=list(), D4=list(), D5=list(), D6=list(), D7=list())
probes = rownames(dataNew[[1]])
for (i in 1:28) for (d in 1:7){
 GE  = mColorh[,i]
 PR  = probes[GE!="grey"]
 dat = dataNew[[d]][GE!="grey",]
 GE  = GE[GE!="grey"]
 dataHM[[d]][[i]] = collapseRows(dat,GE,PR,"MaxMean",FALSE)
 dataHC[[d]][[i]] = collapseRows(dat,GE,PR,"MaxMean",TRUE)
 MEs[[d]][[i]]    = (moduleEigengenes(t(dat), colors= as.character(GE), excludeGrey=TRUE))$eigengenes
};


## Determine all of the expression and connectivity interarray correlations
# This is the section where the data for figure 3A is collected
# Note: you will get a LOT of warnings when running this code.  That is normal.

mat = matrix(nrow=7,ncol=28)
corHME <- corHCE <- corHMC <- corHCC <- corMEs <- list(D1=mat,
  D2=mat, D3=mat, D4=mat, D5=mat, D6=mat, D7=mat)

for (i in 1:28) for (j in 1:7) for (k in 1:7){
  ek = rank(rowSums(dataHM[[k]][[i]][[1]]))
  ej = rank(rowSums(dataHM[[j]][[i]][[1]]))
  corHME[[k]][j,i]=cor(ek,ej)
  ck = rank(softConnectivity(t(dataHM[[k]][[i]][[1]]),
         type="signed", power=9, verbose=0))
  cj = rank(softConnectivity(t(dataHM[[j]][[i]][[1]]),
         type="signed", power=9, verbose=0))
  corHMC[[k]][j,i]=cor(ck,cj)

  ek = rank(rowSums(dataHC[[k]][[i]][[1]]))
  ej = rank(rowSums(dataHC[[j]][[i]][[1]]))
  corHCE[[k]][j,i]=cor(ek,ej)
  ck = rank(softConnectivity(t(dataHC[[k]][[i]][[1]]),
         type="signed", power=9, verbose=0))
  cj = rank(softConnectivity(t(dataHC[[j]][[i]][[1]]),
         type="signed", power=9, verbose=0))
  corHCC[[k]][j,i]=cor(ck,cj)
  ck = rank(softConnectivity(MEs[[k]][[i]],
         type="signed", power=9, verbose=0))
  cj = rank(softConnectivity(MEs[[j]][[i]],
         type="signed", power=9, verbose=0))
  corMEs[[k]][j,i]=cor(ck,cj)
}
# Note, you will get a LOT of warnings in this section.  Ignore them.

pme <- pce <- pmc <- pcc <- pcm <- NULL
for (j in 1:7){
  pme = c(pme, as.numeric(corHME[[j]])); pme = pme[pme<1]
  pmc = c(pmc, as.numeric(corHMC[[j]])); pmc = pmc[pmc<1]
  pce = c(pce, as.numeric(corHCE[[j]])); pce = pce[pce<1]
  pcc = c(pcc, as.numeric(corHCC[[j]])); pcc = pcc[pcc<1]
  pcm = c(pcm, as.numeric(corMEs[[j]])); pcm = pcm[pcm<1]
}
pde=c(pme,pce)
pne=c(rep("1.max",length(pme)),rep("3.kMax",length(pce)))
pdc=c(pmc,pcc,pcm)
pnc=c(rep("1.max",length(pmc)),rep("3.kMax",length(pcc)),
      rep("5.ME",length(pcm)))


## Plot all of the expression and connectivity IACs
# This is the plot for Figure 3A

pdf("genes2ModulesPlot_human_Expr.pdf",height=4.5,width=4)
verboseBarplot(pde,pne,main="Human Brain", xlab="",
  ylab="Pearson Correlation",cex=1.5,ylim=c(0,0.9));  dev.off()
# (Left plot below)
pdf("genes2ModulesPlot_human_Conn.pdf",height=4.5,width=5)
verboseBarplot(pdc,pnc,main="Human Brain",xlab="",
  ylab="Pearson Correlation",cex=1.5,ylim=c(0,0.45)); dev.off()
# (Right plot below)
```

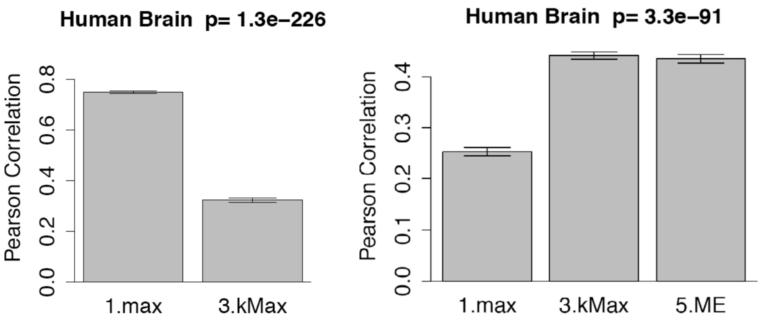


```
## Determine in what percentage of cases each of the methods is best.
# This is the code from which the percentages displayed in figure 3A are formed.

pme <- pce <- pmc <- pcc <- pcm <- NULL
for (j in 1:7){
  pme = c(pme, as.numeric(corHME[[j]]));
  pmc = c(pmc, as.numeric(corHMC[[j]]));
  pce = c(pce, as.numeric(corHCE[[j]]));
  pcc = c(pcc, as.numeric(corHCC[[j]]));
  pcm = c(pcm, as.numeric(corMEs[[j]]));
}
emax = apply(cbind(pme,pce),1,max)
(c(sum(pme==emax),sum(pce==emax))- sum(pme==pce))/(length(emax)- sum(pme==pce))
        # 98% and 2%
cmax = apply(cbind(pmc,pcc,pcm),1,max)
(c(sum(pmc==cmax),sum(pcc==cmax),sum(pcm==cmax))- sum(pmc==pcc))/(length(cmax)- sum(pmc==pcc))
        # 16% and 40% and 44%


################################################################################################################


## Repeat the above analysis looking only at the MOUSE data sets from MGu430A

platM  = c(rep(1,11),2,1,rep(2,7)) # we want "2"
dataNew = dataM[platM==2]
probes = rownames(dataNew[[1]])
for (i in 2:8) probes=intersect(probes,rownames(dataNew[[i]]))
length(probes) # [1] 12851
for (i in 1:8) dataNew[[i]] = dataNew[[i]][probes,]


# Now we have 8 comparable data sets.  The first one will be used as the base set in which modules will be made.
# Comment: In theory, any of these data sets could be used as base.

datExpr1= t(dataNew[[1]])
pickSoftThreshold(datExpr1[,sample(1:12851,7000)],networkType="signed",powerVector=c(9,10,12,14))
#  Power SFT.R.sq slope truncated.R.sq mean.k. median.k. max.k.
#3     9    0.532 -1.69          0.982    89.7      81.6  238.0
#4    10    0.624 -1.73          0.985    65.1      57.5  193.0
#5    12    0.752 -1.79          0.990    36.3      29.9  134.0
#6    14    0.814 -1.91          0.985    21.6      16.3   98.8
# Choose power=12, since R.sq>0.75 and median.k>30 (this data isn't quite as clean as with the human analysis)
# (Due to the sampling of 7000 genes, your results may not be identical. Also, this step above is optional.)

# Note: This section will take a little while (10-45 minutes, probably, and you need a powerful computer)
power = 12
AdjMatrest1 = adjacency(datExpr1,power=power,type="signed");
diag(AdjMatrest1)=0
dissTOM1 = 1-TOMsimilarity(AdjMatrest1, TOMType="signed");
geneTree1 = flashClust(as.dist(dissTOM1),method="average");
collect_garbage()


## Save various module-cutting parameters to use as tests for the collapseRows function.
# Note: This section will take a little while (8-40 minutes, probably)

pdf("collapseRowModuleColorsMouse.pdf", height=30,width=30);
par(mfrow=c(2,1), cex = 1.4, mar = c(0,8.5,2,0));
plot(geneTree1,labels=F,main="Hierarchical dendrogram & module colors",sub="",xlab="")
par(mar = c(1,8.5,0,0));
mColorh=NULL; labels=NULL
```

```
for (ds in 0:3) for (mc in c(4,8,16,32,64,128,256)){
 tree = cutreeHybrid(dendro = geneTree1, pamStage=FALSE,
   minClusterSize = mc, cutHeight = 0.99,
   deepSplit = ds, distM = dissTOM1)
 mColorh=cbind(mColorh,labels2colors(tree$labels));
labels = cbind(labels,paste("Deepsplit =",ds,"; MinSize =",mc))
}
plotHclustColors(geneTree1,mColorh,labels,main = ""); dev.off()
```

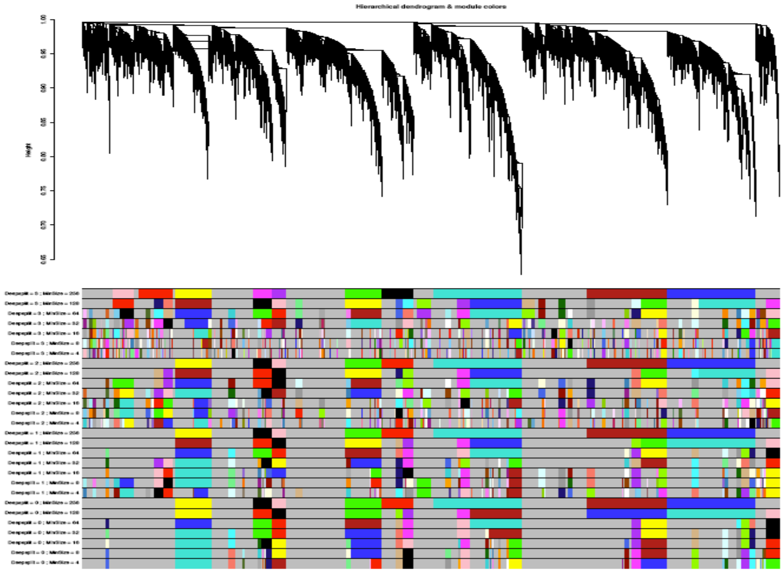

```
## Now run collapseRows a bunch of times, and see which of 3 methods does it best for various module schemas:
# (1) maxMean (refered to as "1.max")
# (2) connectivity method w/ maxMean (refered to as "3.kMax")
# (3) choosing the module eigengene (refered to as "5.ME")
# This step also will take a while (10-45 minutes)

dataMM<-dataMC<-MMEs<-list(D1=list(),D2=list(),D3=list(), D4=list(), D5=list(), D6=list(), D7=list(), D8=list())
probes = rownames(dataNew[[1]])
for (i in 1:28) for (d in 1:8){
 GE  = mColorh[,i]
 PR  = probes[GE!="grey"]
 dat = dataNew[[d]][GE!="grey",]
 GE  = GE[GE!="grey"]
 dataMM[[d]][[i]] = collapseRows(dat,GE,PR,"MaxMean",FALSE)
 dataMC[[d]][[i]] = collapseRows(dat,GE,PR,"MaxMean",TRUE)
 MMEs[[d]][[i]] = (moduleEigengenes(t(dat), colors=
   as.character(GE), excludeGrey=TRUE))$eigengenes
};


## Determine all of the expression and connectivity interarray correlations
# This is the section where the data for figure 3B is collected
# Note: you will get a LOT of warnings when running this code.  That is normal.

mat = matrix(nrow=8,ncol=28)
corMME<-corMCE<-corMMC<-corMCC<-corMMEs<- list(D1=mat, D2=mat, D3=mat, D4=mat, D5=mat, D6=mat, D7=mat, D8=mat)

for (i in 1:28) for (j in 1:8) for (k in 1:8){
  ek = rank(rowSums(dataMM[[k]][[i]][[1]]))
  ej = rank(rowSums(dataMM[[j]][[i]][[1]]))
  corMME[[k]][j,i]=cor(ek,ej)
  ck = rank(softConnectivity(t(dataMM[[k]][[i]][[1]]),
         type="signed", power=9, verbose=0))
  cj = rank(softConnectivity(t(dataMM[[j]][[i]][[1]]),
         type="signed", power=9, verbose=0))
  corMMC[[k]][j,i]=cor(ck,cj)

  ek = rank(rowSums(dataMC[[k]][[i]][[1]]))
  ej = rank(rowSums(dataMC[[j]][[i]][[1]]))
  corMCE[[k]][j,i]=cor(ek,ej)
  ck = rank(softConnectivity(t(dataMC[[k]][[i]][[1]]),
         type="signed", power=9, verbose=0))
  cj = rank(softConnectivity(t(dataMC[[j]][[i]][[1]]),
         type="signed", power=9, verbose=0))
  corMCC[[k]][j,i]=cor(ck,cj)
```

```
  ck = rank(softConnectivity(MMEs[[k]][[i]],
         type="signed", power=9, verbose=0))
  cj = rank(softConnectivity(MMEs[[j]][[i]],
         type="signed", power=9, verbose=0))
  corMMEs[[k]][j,i]=cor(ck,cj)
}

pme <- pce <- pmc <- pcc <- pcm <- NULL
for (j in 1:7){
  pme = c(pme, as.numeric(corMME[[j]])); pme = pme[pme<1]
  pmc = c(pmc, as.numeric(corMMC[[j]])); pmc = pmc[pmc<1]
  pce = c(pce, as.numeric(corMCE[[j]])); pce = pce[pce<1]
  pcc = c(pcc, as.numeric(corMCC[[j]])); pcc = pcc[pcc<1]
  pcm = c(pcm, as.numeric(corMMEs[[j]])); pcm = pcm[pcm<1]
}
# Note: This section will produce a lot of warnings.  Ignore them.

pde=c(pme,pce)
pne=c(rep("1.max",length(pme)),rep("3.kMax",length(pce)))
pdc=c(pmc,pcc,pcm)
pnc=c(rep("1.max",length(pmc)),rep("3.kMax",length(pcc)),
      rep("5.ME",length(pcm)))


## Plot all of the expression and connectivity IACs
# This is the plot for Figure 3B

pdf("genes2ModulesPlot_mouse_Expr.pdf",height=4.5,width=4)
verboseBarplot(pde,pne,main="Mouse Brain", xlab="",
  ylab="Pearson Correlation",cex=1.5,ylim=c(0,0.9));  dev.off()
pdf("genes2ModulesPlot_mouse_Conn.pdf",height=4.5,width=5)
verboseBarplot(pdc,pnc,main="Mouse Brain",xlab="",
  ylab="Pearson Correlation",cex=1.5,ylim=c(0,0.45)); dev.off()
```

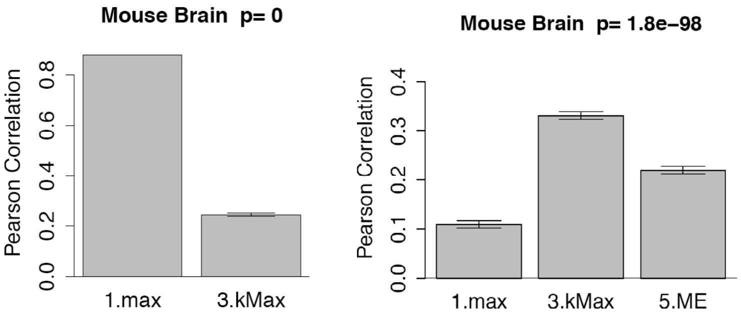


```
## Determine in what percentage of cases each of the methods is best.
# This is the code from which the percentages displayed in figure 3B are formed.

pme <- pce <- pmc <- pcc <- pcm <- NULL
for (j in 1:7){
  pme = c(pme, as.numeric(corMME[[j]]));
  pmc = c(pmc, as.numeric(corMMC[[j]]));
  pce = c(pce, as.numeric(corMCE[[j]]));
  pcc = c(pcc, as.numeric(corMCC[[j]]));
  pcm = c(pcm, as.numeric(corMMEs[[j]]));
}
emax = apply(cbind(pme,pce),1,max);
(c(sum(pme==emax),sum(pce==emax))- sum(pme==pce))/(length(emax)- sum(pme==pce))
       # 100% and 0%
cmax = apply(cbind(pmc,pcc,pcm),1,max);
(c(sum(pmc==cmax),sum(pcc==cmax),sum(pcm==cmax))- sum(pmc==pcc))/(length(cmax)- sum(pmc==pcc))
       # 15% and 53% and 32%
```

```
################################
## CODE FOR MAKING FIGURE 3C ##
################################

# Load and properly format the data
# Note: since these data are from multiple array platforms, collapseRows has already been performed to collapse
probes to genes.

load("dataForFigure3C.RData")
dataNew      = list();        dataNew[[1]] = datExpr1;      dataNew[[2]] = datExpr2
dataNew[[3]] = datExpr3;      dataNew[[4]] = datExpr4;      dataNew[[5]] = datExpr5
probes       = rownames(dataNew[[1]])
for (i in 2:5) probes=intersect(probes,rownames(dataNew[[i]]))
length(probes) # [1] 11163
for (i in 1:5) dataNew[[i]] = dataNew[[i]][probes,]


# Now we have 5 comparable data sets.  The first one will be used as the base set in which modules will be made.
# Comment: In theory, any of these data sets could be used as base.

datExpr1= t(dataNew[[1]])
pickSoftThreshold(datExpr1[,sample(1:11163,7000)],networkType="signed",powerVector=c(6,8,9,10,12))
# Power    SFT.R.sq     slope truncated.R.sq    mean.k. median.k.    max.k.
# 1     6 0.8953384 -5.275143      0.9578619 143.871439 134.32663 273.66126
# 2     8 0.8907455 -3.577839      0.9682040  46.467893  39.97028 134.36925
# 3     9 0.8921945 -3.073353      0.9757536  27.519645  22.24764  98.60808
# 4    10 0.8763925 -2.762219      0.9781986  16.798939  12.53478  74.10556
# 5    12 0.8490385 -2.325980      0.9791452   6.880352   4.12069  44.20459
# Choose power=9, to try and balance the slope, R.sq, and median.k results (power=6-10 would also have worked)
#  (Due to the sampling of 7000 genes, your results may not be identical. Also, this step above is optional.)

# Note: This section will take a little while (5-45 minutes, probably, and you need a powerful computer)
power = 9
AdjMatrest1 = adjacency(datExpr1,power=power,type="signed");
diag(AdjMatrest1)=0
dissTOM1 = 1-TOMsimilarity(AdjMatrest1, TOMType="signed");
geneTree1 = flashClust(as.dist(dissTOM1),method="average");
collectGarbage()


## Save various module-cutting parameters to use as tests for the collapseRows function.
# Note: This section will take a little while (5-30 minutes, probably)

pdf("collapseRowModuleColorsHumanBlood.pdf", height=30,width=30);
par(mfrow=c(2,1), cex = 1.4, mar = c(0,8.5,2,0));
plot(geneTree1,labels=F,main="Hierarchical dendrogram & module colors",sub="",xlab="")
par(mar = c(1,8.5,0,0));
mColorh=NULL; labels=NULL
for (ds in 0:3) for (mc in c(4,8,16,32,64,128,256)){
 tree = cutreeHybrid(dendro = geneTree1, pamStage=FALSE,
   minClusterSize = mc, cutHeight = 0.99,
   deepSplit = ds, distM = dissTOM1)
 mColorh=cbind(mColorh,labels2colors(tree$labels));
labels = cbind(labels,paste("Deepsplit =",ds,"; MinSize =",mc))
}
plotHclustColors(geneTree1,mColorh,labels,main = "");
dev.off()
```

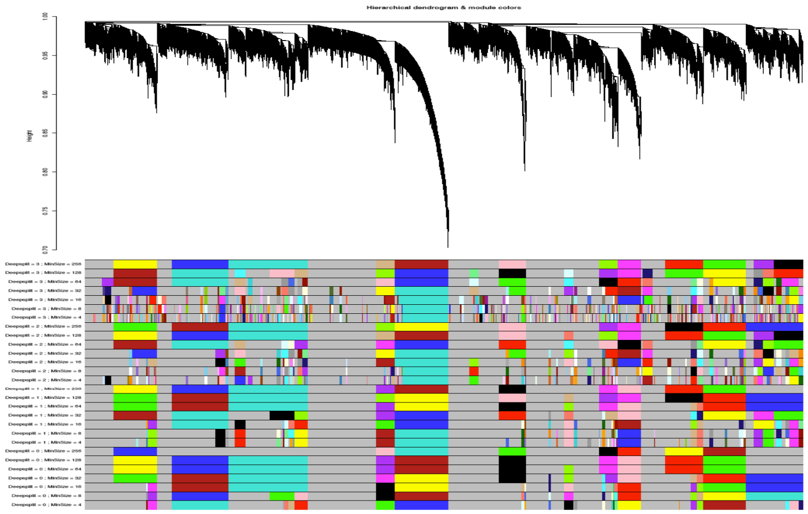

```
## Now run collapseRows a bunch of times, and see which of 3 methods does it best for various module schemas:
# (1) maxMean (refered to as "1.max")
# (2) connectivity method w/ maxMean (refered to as "3.kMax")
# (3) choosing the module eigengene (refered to as "5.ME")
# This step also will take a while (10-45 minutes)

dataHM<-dataHC<-MEs<-list(D1=list(),D2=list(),D3=list(), D4=list(), D5=list())
probes = rownames(dataNew[[1]])
for (i in 1:28) for (d in 1:5){
 GE  = mColorh[,i]
 PR  = probes[GE!="grey"]
 dat = dataNew[[d]][GE!="grey",]
 GE  = GE[GE!="grey"]
 dataHM[[d]][[i]] = collapseRows(dat,GE,PR,"MaxMean",FALSE)
 dataHC[[d]][[i]] = collapseRows(dat,GE,PR,"MaxMean",TRUE)
 MEs[[d]][[i]]    = (moduleEigengenes(t(dat), colors= as.character(GE), excludeGrey=TRUE))$eigengenes
};


## Determine all of the expression and connectivity interarray correlations
# This is the section where the data for figure 3C is collected
# Note: you will get a LOT of warnings when running this code.  That is normal.

mat = matrix(nrow=5,ncol=28)
corHME <- corHCE <- corHMC <- corHCC <- corMEs <- list(D1=mat, D2=mat, D3=mat, D4=mat, D5=mat)

for (i in 1:28) for (j in 1:5) for (k in 1:5){
  ek = rank(rowSums(dataHM[[k]][[i]][[1]]))
  ej = rank(rowSums(dataHM[[j]][[i]][[1]]))
  corHME[[k]][j,i]=cor(ek,ej)
  ck = rank(softConnectivity(t(dataHM[[k]][[i]][[1]]),
         type="signed", power=9, verbose=0))
  cj = rank(softConnectivity(t(dataHM[[j]][[i]][[1]]),
         type="signed", power=9, verbose=0))
  corHMC[[k]][j,i]=cor(ck,cj)

  ek = rank(rowSums(dataHC[[k]][[i]][[1]]))
  ej = rank(rowSums(dataHC[[j]][[i]][[1]]))
  corHCE[[k]][j,i]=cor(ek,ej)
  ck = rank(softConnectivity(t(dataHC[[k]][[i]][[1]]),
         type="signed", power=9, verbose=0))
  cj = rank(softConnectivity(t(dataHC[[j]][[i]][[1]]),
         type="signed", power=9, verbose=0))
  corHCC[[k]][j,i]=cor(ck,cj)
  ck = rank(softConnectivity(MEs[[k]][[i]],
         type="signed", power=9, verbose=0))
  cj = rank(softConnectivity(MEs[[j]][[i]],
         type="signed", power=9, verbose=0))
  corMEs[[k]][j,i]=cor(ck,cj)
}
# Note, you will get a LOT of warnings in this section.  Ignore them.

pme <- pce <- pmc <- pcc <- pcm <- NULL
for (j in 1:5){
  pme = c(pme, as.numeric(corHME[[j]])); pme = pme[pme<1]
  pmc = c(pmc, as.numeric(corHMC[[j]])); pmc = pmc[pmc<1]
  pce = c(pce, as.numeric(corHCE[[j]])); pce = pce[pce<1]
  pcc = c(pcc, as.numeric(corHCC[[j]])); pcc = pcc[pcc<1]
  pcm = c(pcm, as.numeric(corMEs[[j]])); pcm = pcm[pcm<1]
}

pde=c(pme,pce)
pne=c(rep("1.max",length(pme)),rep("3.kMax",length(pce)))
pdc=c(pmc,pcc,pcm)
pnc=c(rep("1.max",length(pmc)),rep("3.kMax",length(pcc)),
      rep("5.ME",length(pcm)))


## Plot all of the expression and connectivity IACs
# This is the plot for Figure 3C

pdf("genes2ModulesPlot_human_Blood_Expr.pdf",height=4.5,width=4)
verboseBarplot(pde,pne,main="Human Blood", xlab="",
  ylab="Pearson Correlation",cex=1.5,ylim=c(0,0.9));  dev.off()
# (Left plot below)
pdf("genes2ModulesPlot_human_Blood_Conn.pdf",height=4.5,width=5)
verboseBarplot(pdc,pnc,main="Human Blood",xlab="",
  ylab="Pearson Correlation",cex=1.5,ylim=c(0,0.45)); dev.off()
# (Right plot below)
```

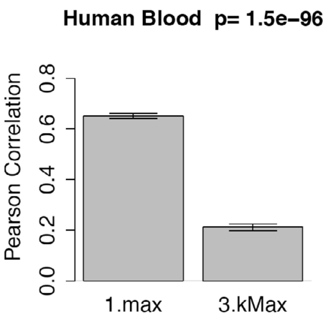

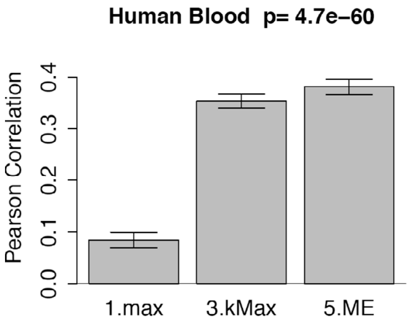


```
## Determine in what percentage of cases each of the methods is best.
# This is the code from which the percentages displayed in figure 3C are formed.

pme <- pce <- pmc <- pcc <- pcm <- NULL
for (j in 1:5){
  pme = c(pme, as.numeric(corHME[[j]]));
  pmc = c(pmc, as.numeric(corHMC[[j]]));
  pce = c(pce, as.numeric(corHCE[[j]]));
  pcc = c(pcc, as.numeric(corHCC[[j]]));
  pcm = c(pcm, as.numeric(corMEs[[j]]));
}
emax = apply(cbind(pme,pce),1,max)
(c(sum(pme==emax),sum(pce==emax))- sum(pme==pce))/(length(emax)- sum(pme==pce))
        # 89% and 11%
cmax = apply(cbind(pmc,pcc,pcm),1,max)
(c(sum(pmc==cmax),sum(pcc==cmax),sum(pcm==cmax))- sum(pmc==pcc))/(length(cmax)- sum(pmc==pcc))
        # 14% and 34% and 52%
```

```
##############################
## CODE FOR MAKING FIGURE 4 ##
##############################

## Here we use the collapseRows function to predict the relative quantities of blood cell lines across mixed
samples, given a set of pure cell lines from which we can first find marker genes.

## First load the data
# Note: This includes the expression data, variables marking cell types (1=yes, 2=no), and sample information.

load("abbas_start.RData")
attach(datSample)

## Use the standard screening to find marker genes for each cell type based on fold change
# Note: This step takes a few minutes (~5-15)

ResultIM9    = standardScreeningBinaryTrait(datExpr, y=yIM9)
ResultJurkat = standardScreeningBinaryTrait(datExpr, y=yJurkat)
ResultRaji   = standardScreeningBinaryTrait(datExpr, y=yRaji)
ResultTHP1   = standardScreeningBinaryTrait(datExpr, y=yTHP1)

numberMarkerGenesForEachCell = 500
selectGeneIM9    = rank(-ResultIM9$FoldChange.1.vs.2, ties.method="first")<=numberMarkerGenesForEachCell
selectGeneJurkat = rank(-ResultJurkat$FoldChange.1.vs.2, ties.method="first")<=numberMarkerGenesForEachCell
selectGeneRaji   = rank(-ResultRaji$FoldChange.1.vs.2, ties.method="first")<=numberMarkerGenesForEachCell
selectGeneTHP1   = rank(-ResultTHP1$FoldChange.1.vs.2, ties.method="first")<=numberMarkerGenesForEachCell
selectGene       = selectGeneJurkat | selectGeneIM9  | selectGeneRaji | selectGeneTHP1


## Remember we know the following true mixture proportions.  We need to scale it such that the columns (cell
types), rather than the rows scale to 1.
# This is what we compare our results against in figure 3.
# Note that this is the step that removes the calibration (ie, we can no longer predict the absolute
proportions, only the relative proportions)

selectSamplesForCollapse = is.element(Population,c("MixA","MixB","MixC","MixD") )
datExprForCollapse       = datExpr[ selectSamplesForCollapse, ]
datTrueProportions       = datSample[ selectSamplesForCollapse,  c(6,5,7,8) ]
datTrueProportionsCT     = datTrueProportions
for (i in 1:4) datTrueProportionsCT[,i] = datTrueProportionsCT[,i]/sum(datTrueProportionsCT[,i])


## Next we need to make a vector corresponding to module (ie., "group")

whichCellType = rep("grey",dim(datExprForCollapse)[2])
names(whichCellType) = colnames(datExprForCollapse)
whichCellType[selectGeneJurkat] = "Jurkat"
whichCellType[selectGeneIM9]    = "IM9"
whichCellType[selectGeneRaji]   = "Raji"
whichCellType[selectGeneTHP1]   = "THP1"


## Next we need to run collapseRows using multiple parameter choices
# (1) maxMean (refered to as "1.max")
# (2) connectivity method w/ maxMean (refered to as "3.kMax")
# (3) choosing the module eigengene (refered to as "5.ME")
# (4) taking the average of all genes (refered to as "6.Avg")

# source("collapseRows_04_11_11.R")  ## NOT NECESSARY IF YOUR WGCNA LIBRARY IS UP TO DATE ##
datExprCR  = t(datExprForCollapse[,whichCellType!="grey"])
geneID     = colnames(datExprForCollapse)
predictMax = t(collapseRows(datExprCR, whichCellType, geneID, method="MaxMean",
             connectivityBasedCollapsing=FALSE)[[1]])
predictCon = t(collapseRows(datExprCR, whichCellType, geneID, method="MaxMean",
             connectivityBasedCollapsing=TRUE)[[1]])
predictME  = t(collapseRows(datExprCR, whichCellType, geneID, method="ME")[[1]])
predictAvg = t(collapseRows(datExprCR, whichCellType, geneID, method="function",
             connectivityBasedCollapsing=FALSE, methodFunction=colMeans)[[1]])


## Next we need to scale each of the predicted results to 1, so they match the actual results

for (i in 1:4){
  predictMax[,i] = predictMax[,i]/sum(predictMax[,i])
  predictCon[,i] = predictCon[,i]/sum(predictCon[,i])
  # NOTE, we do not need to scale the ME method, since by definition, the ME pre-scales the sum to 0
  predictAvg[,i] = predictAvg[,i]/sum(predictAvg[,i])
}
```

```
## Now lets make the actual vs. predicted plots for all four methods.
## These are the actual plots for Figure 4

pdf("fourMethod_actual_vs_predicted_results_Abbas.pdf",width=5,height=5.5)
verboseScatterplot(as.matrix(predictMax)[1:48],  as.matrix(datTrueProportionsCT)[1:48], pch=19,
  xlab="Predicted proportion", ylab="true proportion",main="1.Max estimation");   abline(0,1);
verboseScatterplot(as.matrix(predictCon)[1:48],  as.matrix(datTrueProportionsCT)[1:48], pch=19,
  xlab="Predicted proportion", ylab="true proportion",main="3.kMax estimation");  abline(0,1);
verboseScatterplot(as.matrix(predictME)[1:48],   as.matrix(datTrueProportionsCT)[1:48], pch=19,
  xlab="Predicted proportion", ylab="true proportion",main="5.ME estimation");            # NO ABLINE
verboseScatterplot(as.matrix(predictAvg)[1:48],  as.matrix(datTrueProportionsCT)[1:48], pch=19,
  xlab="Predicted proportion", ylab="true proportion",main="6.Avg estimation");   abline(0,1)
dev.off()
```

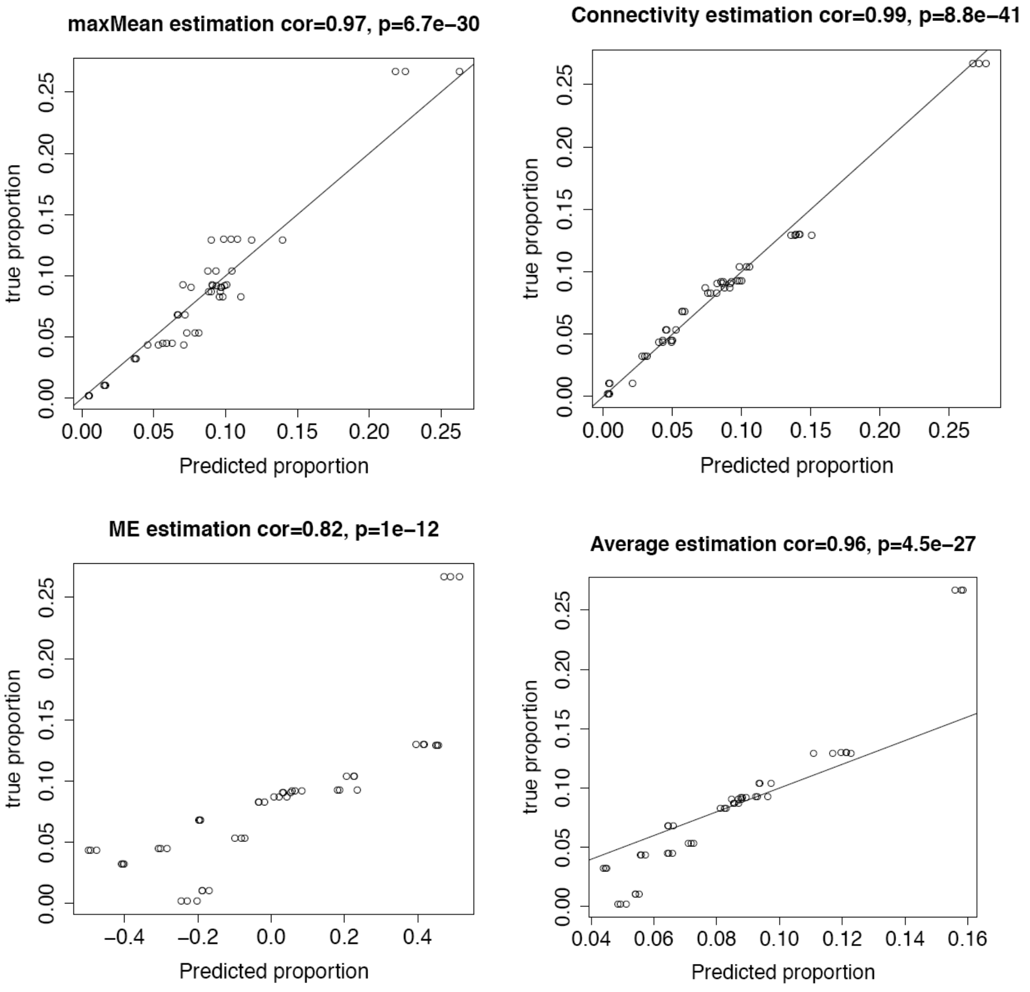

```
###############################
## CODE FOR MAKING FIGURE 5 ##
###############################

## Here we use the collapseRows function to predict the relative quantities of blood cell types across whole
blood, given a set of pure cell types from which we can first find marker genes, and given CBC values (known
proportions based on flow cytometry).

## First load the data
# Note: This includes the expression data and a data variable containing sample information.

load("Grigoryev_start.RData")
attach(datSample)
library(WGCNA)


## Choose the cell-type-specific genes as the ones with highest expression in that cell type relative to the
cell type with the second highest average expression
# Note: this is because using direct fold change chooses genes that are markers for multiple cell types, which
does not work with collapseRows.

dat0        = 2^datExpr[Time==0,]           # The data is in log2 space.
rownames(datSample) = datSample$SampleGSMID
nas         = rowSums(is.na(datSample))      # Omit all samples with missing data
sampleWB    = t(datSample[nas<=1,])
sampleWB    = sampleWB[rowSums(is.na(sampleWB))==0,]
CellSubType = as.character(CellSubType)
names(CellSubType) <- names(Time) <- rownames(datExpr)

cst0 = as.character(CellSubType[Time==0])
cellTypes = names(table(cst0))
inReg <- bestOutReg <- matrix(0,nrow=dim(dat0)[2],ncol = length(cellTypes))
colnames(inReg) <- colnames(bestOutReg) <- cellTypes
for (c in cellTypes)  inReg[,c] = colMeans(dat0[cst0==c,])                 # Mean expression in cell type
for (c in cellTypes)  bestOutReg[,c] = apply(inReg[,cellTypes!=c],1,max)   # Mean expr. of next best cell type.


## Find the top marker genes for various numbers of markers

rankCTmarkers = bestOutReg*0
for (c in cellTypes)
 rankCTmarkers[,c] = rank(-inReg[,c]/ bestOutReg[,c])

whichCellType = list()        # This is the "group" variable for collapseRows
numMarkers    = c(5, 20, 50, 100, 200, 500)
for (i in 1:length(numMarkers)){
 whichCellType[[i]] = rep("grey", dim(datExpr)[2])
 for (c in cellTypes)  whichCellType[[i]][rankCTmarkers[,c]<=numMarkers[i]]=c
}


## Next we need to run collapseRows using multiple parameter choices

# source("collapseRows_04_11_11.R") ## NOT NECESSARY IF YOUR WGCNA LIBRARY IS UP TO DATE ##
datWB = 2^datExpr[colnames(sampleWB),]       # The data is in log2 space.
geneID     = colnames(datWB)

predictMax <- predictCon <- predictME <- predictAvg <- list()
for (i in 1:length(numMarkers)){
 datExprCR = t(datWB[,whichCellType[[i]]!="grey"])
 predictMax[[i]] = t(collapseRows(datExprCR, whichCellType[[i]], geneID, method="MaxMean",
   connectivityBasedCollapsing=FALSE)[[1]])
 predictCon[[i]] = t(collapseRows(datExprCR, whichCellType[[i]], geneID, method="MaxMean",
   connectivityBasedCollapsing=TRUE)[[1]])
 predictME[[i]]  = t(collapseRows(datExprCR, whichCellType[[i]], geneID, method="ME")[[1]])
 predictAvg[[i]] = t(collapseRows(datExprCR, whichCellType[[i]], geneID, method="Average",
   connectivityBasedCollapsing=FALSE)[[1]])
}
# Note: This code will make a lot of warnings.  You can ignore these.
```

## See how well the actual and predicted counts agree, for each method and each set of marker genes.

```
# This function tells us the average actual-vs.-predicted correlation across all cell types tested
meanCorrCorrect = function(actual, vector, predict){
  out     = NULL;
  results = cor(actual, predict, use="p")
  for (i in 1:length(vector))  out = c(out, results[i,vector[i]])
  return(mean(out))
}

count3 = t(sampleWB[c("RATIO.BcellsdividedByWBC","RATIO.CD4TcellsdividedByWBC","RATIO.CD8TcellsdividedByWBC"),])
    # This variable represents the true proportion of each cell type tested.  Since CD19, CD4, and CD8 are the
    only cell types that can be accurately predicted using deconvolution (not shown), we only try and predict
    the proportions of these cells using collapseRows as well.  Note that we use the ratio of each cell type
    divided by whole blood, rather than a cell count.  This takes the place of the normalization process from
    the previous analysis.

checkCol = c(2,3,5) # Corresponding to CD19, CD4, and CD8, respectively
meanCorrect = matrix(0, nrow = 4, ncol = length(numMarkers))
rownames(meanCorrect) = c("MaxMean","Connectivity","ME","Average")
colnames(meanCorrect) = as.character(numMarkers)
for (i in 1:length(numMarkers)){
 meanCorrect[1,i] = meanCorrCorrect(count3,checkCol,predictMax[[i]])
 meanCorrect[2,i] = meanCorrCorrect(count3,checkCol,predictCon[[i]])
 meanCorrect[3,i] = meanCorrCorrect(count3,checkCol,predictME[[i]])
 meanCorrect[4,i] = meanCorrCorrect(count3,checkCol,predictAvg[[i]])
}; meanCorrect

#############################################################################
# Method               5         20         50        100        200        500 #
# MaxMean      0.3226094 0.5034603 0.3771062 0.5574753 0.3666298 0.2817615 #
# Connectivity 0.5342018 0.4615812 0.6209047 0.6209047 0.5103112 0.4898451 #
# ME           0.6267194 0.5265878 0.5842563 0.5860148 0.5437780 0.4433879 #
# Average      0.3939926 0.4997936 0.5527694 0.6048880 0.5990240 0.5673713 #
#############################################################################
```

## Now plot these results as a scatterplot

```
pdf("Figure_5.pdf",height=4,width=8)
datCor = as.data.frame(t(meanCorrect))
matplot(numMarkers, datCor,type="b",log="x",main="Corrrelation between actual and predicted values",
  ylab="Correlation (R)",xlab="Number of marker genes")
legend(10,0.4,colnames(datCor),pch="1234",col=c("black","red","green","blue"),
  text.col=c("black","red","green","blue"),cex=0.7)
dev.off()
```

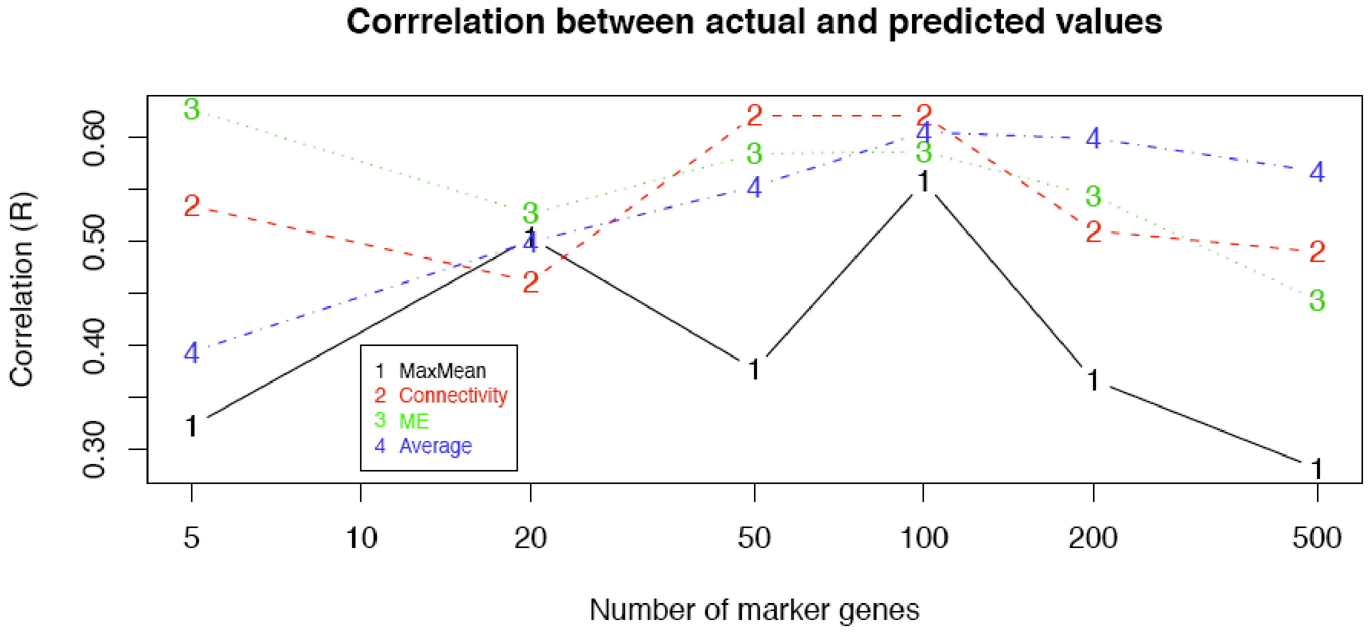

```
#######################################
## CODE FOR MAKING ADDITIONAL FILE 3 ##
#######################################

## Get the subset of samples / probes that we want for this analysis

# Find the human data sets from HGu133A
platH  = c(2,2,1,3,3,3,3,3,3,2,2,3,1,2,2,2,3,2) # we want "2" (HGu133A)
dataH_u133A = dataH[platH==2][2:8][c(5,6,2,3,4,7,1)] # (as above)
probesH = rownames(dataH_u133A[[1]])
for (i in 2:7) probesH=intersect(probesH,rownames(dataH_u133A[[i]]))
length(probesH) # [1] 10306
for (i in 1:7) dataH_u133A[[i]] = dataH_u133A[[i]][probesH,]

# Find the mouse data sets from MGU430A
platM  = c(rep(1,11),2,1,rep(2,7)) # we want "2" (MGu430A)
dataM_u430A = dataM[platM==2]
probesM = rownames(dataM_u430A[[1]])
for (i in 2:8) probesM=intersect(probesM,rownames(dataM_u430A[[i]]))
length(probesM) # [1] 12851
for (i in 1:8) dataM_u430A[[i]] = dataM_u430A[[i]][probesM,]

# Omit all genes that only have 1 probe, since these will be the same in both cases
kp133 <- kp430 <- NULL
kp133 = subsetDat(dataH_u133A[[1]],GE,PR,2)[[3]]
kp430 = subsetDat(dataM_u430A[[1]],GE,PR,2)[[3]]
for (i in 1:7) dataH_u133A[[i]] = dataH_u133A[[i]][kp133,]
for (i in 1:8) dataM_u430A[[i]] = dataM_u430A[[i]][kp430,]

## Run collapseRows

dataH_u133A_mm <- dataM_u430A_mm <- list()
for (i in 1:7)
 dataH_u133A_mm[[i]] = collapseRows(dataH_u133A[[i]],GE,PR,"MaxMean")
for (i in 1:8)
 dataM_u430A_mm[[i]] = collapseRows(dataM_u430A[[i]],GE,PR,"MaxMean")

## Get the correlation information

rnkExprH <- rnkExprH_mm <- rnkExprM <- rnkExprM_mm <- list()
for (i in 1:7) rnkExprH[[i]]    = rank(rowSums(dataH_u133A[[i]]))
for (i in 1:7) rnkExprH_mm[[i]] = rank(rowSums(dataH_u133A_mm[[i]][[1]]))
for (i in 1:8) rnkExprM[[i]]    = rank(rowSums(dataM_u430A[[i]]))
for (i in 1:8) rnkExprM_mm[[i]] = rank(rowSums(dataM_u430A_mm[[i]][[1]]))

corH <- corH_mm <- matrix(nrow=7,ncol=7)
for (i in 1:7) for (j in 1:7){
  ci = rnkExprH[[i]];      cj = rnkExprH[[j]];      ov = intersect(names(ci),names(cj))
  corH[i,j]    = cor(rank(ci[ov]),rank(cj[ov]))
  ci = rnkExprH_mm[[i]];   cj = rnkExprH_mm[[j]];   ov = intersect(names(ci),names(cj))
  corH_mm[i,j] = cor(rank(ci[ov]),rank(cj[ov]))
}
corM <- corM_mm <- matrix(nrow=8,ncol=8)
for (i in 1:8) for (j in 1:8){
  ci = rnkExprM[[i]];      cj = rnkExprM[[j]];      ov = intersect(names(ci),names(cj))
  corM[i,j]    = cor(rank(ci[ov]),rank(cj[ov]))
  ci = rnkExprM_mm[[i]];   cj = rnkExprM_mm[[j]];   ov = intersect(names(ci),names(cj))
  corM_mm[i,j] = cor(rank(ci[ov]),rank(cj[ov]))
}

## Get the connectivity information

rnkConnH <- rnkConnM <- rnkConnH_mm <- rnkConnM_mm <- list()
for (i in 1:7){
  dat = t(dataH_u133A[[i]])
  rnkConnH[[i]] = rank(softConnectivity(dat, type="signed", power=10, verbose=0))
  names(rnkConnH[[i]]) = colnames(dat)
  dat = t(dataH_u133A_mm[[i]][[1]])
  rnkConnH_mm[[i]] = rank(softConnectivity(dat, type="signed", power=10, verbose=0))
  names(rnkConnH_mm[[i]]) = colnames(dat)
}
for (i in 1:8){
  dat = t(dataM_u430A[[i]])
  rnkConnM[[i]] = rank(softConnectivity(dat, type="signed", power=10, verbose=0))
  names(rnkConnM[[i]]) = colnames(dat)
  dat = t(dataM_u430A_mm[[i]][[1]])
  rnkConnM_mm[[i]] = rank(softConnectivity(dat, type="signed", power=10, verbose=0))
  names(rnkConnM_mm[[i]]) = colnames(dat)
}
```

```
corCH <- corCH_mm <- matrix(nrow=7,ncol=7)
for (i in 1:7) for (j in 1:7){
  ci = rnkConnH[[i]];      cj = rnkConnH[[j]];      ov = intersect(names(ci),names(cj))
  corCH[i,j]    = cor(rank(ci[ov]),rank(cj[ov]))
  ci = rnkConnH_mm[[i]];   cj = rnkConnH_mm[[j]];   ov = intersect(names(ci),names(cj))
  corCH_mm[i,j] = cor(rank(ci[ov]),rank(cj[ov]))
}
corCM <- corCM_mm <- matrix(nrow=8,ncol=8)
for (i in 1:8) for (j in 1:8){
  ci = rnkConnM[[i]];      cj = rnkConnM[[j]];      ov = intersect(names(ci),names(cj))
  corCM[i,j]    = cor(rank(ci[ov]),rank(cj[ov]))
  ci = rnkConnM_mm[[i]];   cj = rnkConnM_mm[[j]];   ov = intersect(names(ci),names(cj))
  corCM_mm[i,j] = cor(rank(ci[ov]),rank(cj[ov]))
}

## Make the plots

pdf("Additional_File_3.pdf",width=9,height=9)
par(mfrow=c(2,2))
l = sum(lower.tri(corH));
dNames = c(rep("0.None",l), rep("1.max",l))
d=c(corH_mm[lower.tri(corH_mm)], corH[lower.tri(corH)])
verboseBarplot(d,dNames,main="Human Brain",xlab="",ylab="Reproducibility (Correlation)",cex=1.5)
d=c(corCH_mm[lower.tri(corCH_mm)], corCH[lower.tri(corCH)])
verboseBarplot(d,dNames,main="Human Brain",xlab="",ylab="Reproducibility (Correlation)",cex=1.5)

l = sum(lower.tri(corM));
dNames = c(rep("0.None",l), rep("1.max",l))
d=c(corM_mm[lower.tri(corM_mm)], corM[lower.tri(corM)])
verboseBarplot(d,dNames,main="Mouse Brain",xlab="",ylab="Reproducibility (Correlation)",cex=1.5)
d=c(corCM_mm[lower.tri(corCM_mm)], corCM[lower.tri(corCM)])
verboseBarplot(d,dNames,main="Mouse Brain",xlab="",ylab="Reproducibility (Correlation)",cex=1.5)
dev.off()
```

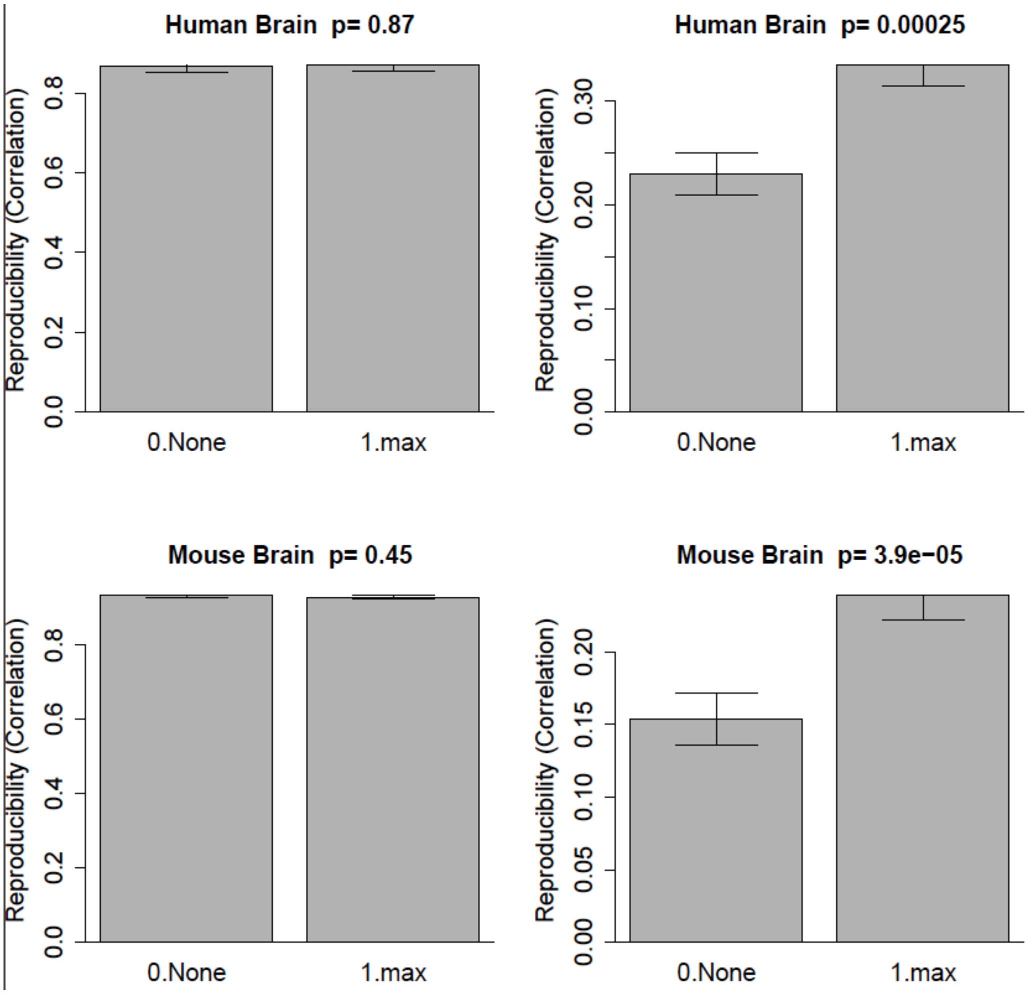

Supplement: Additional file 2 — R code to perform analysis. This file includes all of the code required to reproduce Figures 2, 3, 4, 5 in this manuscript (and Additional file 3 - Increase in reproducibility using collapseRows), along with a limited amount of annotation. Data for use with this code is available at the collapseRows website [6]. [file 1471-2105-12-322-S2.PDF]
