# Supplementary material for: Strategies for aggregating gene expression data: The collapseRows R function
Source: BMC Bioinformatics. 2011 Aug 4;12:322. doi: 10.1186/1471-2105-12-322 (PMC3166942; doi:10.1186/1471-2105-12-322)
Supplement: Additional file 3 — Increase in reproducibility using collapseRows. We calculated ranked expression (left column) and ranked connectivity (right column) correlation across 7 studies in human brain run on the HGu133A platform (A) and 8 studies in mouse brain run on the MGu430A platform (B). To assess change in reproducibility due to collapseRows, we compared these correlations using our best collapsing method (1.max) against uncollapsed data (0.None). We do not find any changes in reproducibility based on expression correlation; however, based on connectivity correlation we find a relatively substantial increase in reproducibility in both species. Note that these correlations are generally higher than the corresponding correlations from Figure 2 because we only show the correlations from data sets coming from the same platform. Y-axes correspond to the average expression and connectivity correlation between data sets. Error bars represent standard error. [file 1471-2105-12-322-S3.PDF]

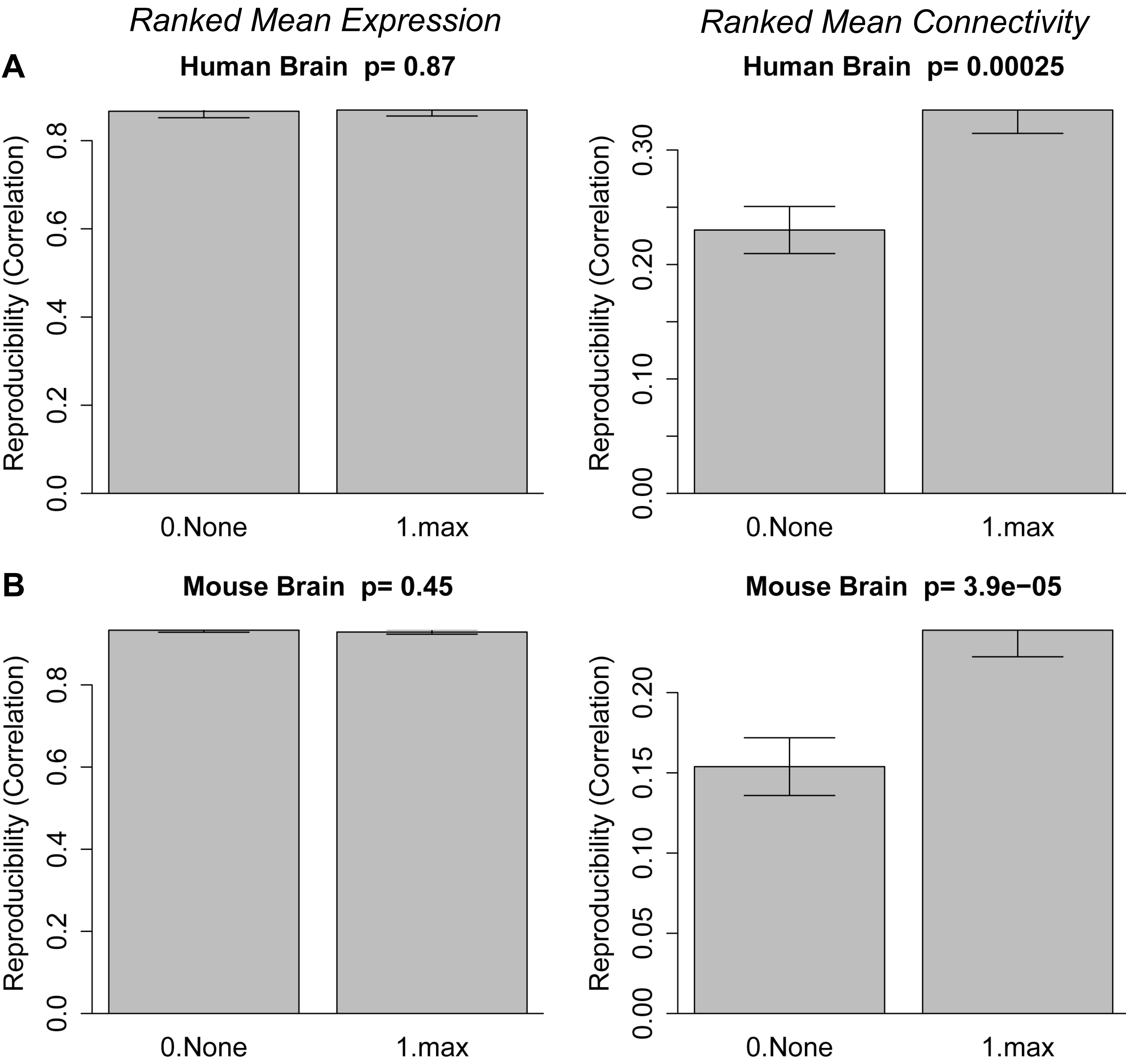

Ranked Mean Expression
Ranked Mean Connectivity
A
Human Brain p= 0.87
Human Brain p= 0.00025
Reproducibility (Correlation)
0.0
0.2
0.4
0.6
0.8
0.00
0.10
0.20
0.30
0.None
1.max
B
Mouse Brain p= 0.45
Mouse Brain p= 3.9e−05
0.00
0.05
0.10
0.15
0.20
